# Effectiveness of home-based pulmonary rehabilitation programs for patients with chronic obstructive pulmonary disease (COPD): systematic review

**DOI:** 10.1186/s12913-022-07779-9

**Published:** 2022-04-26

**Authors:** Tania Stafinski, Fernanda Inagaki Nagase, Melita Avdagovska, Michael K. Stickland, Devidas Menon

**Affiliations:** 1 School of Public Health, Health Technology and Policy Unit, 3-021 Research Transition Facility, University of Alberta, Edmonton, Alberta T6G 2V2 Canada; 2 Alberta Health Services, Edmonton, Alberta Canada; 3 Division of Pulmonary Medicine, Faculty of Medicine & Dentistry, University of Alberta, Edmonton, Alberta Canada; 4 G.F. MacDonald Centre for Lung Health, Covenant Health, Edmonton, Alberta Canada

**Keywords:** COPD, Pulmonary rehabilitation, Home-based, Systematic review

## Abstract

**Background:**

Although pulmonary rehabilitation (PR) is considered a key component in managing chronic obstructive pulmonary disease (COPD) patients, uptake remains suboptimal. This systematic review aimed to determine the effectiveness of home-based PR (HBPR) programs for COPD patients.

**Methods:**

A systematic review of scholarly literature published within the last 10 years from the conception of this project was conducted using internationally recognized guidelines. Search strategies were applied to electronic databases and clinical trial registries through March 2020 and updated in November 2021 to identify studies comparing HBPR with ‘usual care’ or outpatient pulmonary rehabilitation (OPR). To critically appraise randomized studies, the Cochrane Collaboration risk of bias tool (ROB) was used. The quality of non-randomized studies was evaluated using the ACROBAT-NRSI tool. The quality of evidence relating to key outcomes was assessed using Grading of Recommendations, Assessment, Development and Evaluations (GRADE) on health-related quality of life (HRQoL), exacerbation frequencies, COPD-related hospital admissions, and program adherence. Three independent reviewers assessed methodologic quality and reviewed the studies.

**Results:**

Twelve randomized controlled trials (RCTs) and 2 comparative observational studies were included. While considerable evidence relating to the effectiveness of HBPR programs for COPD patients exist, overall quality is low. There were no differences between HBPR and OPR in terms of safety, HRQoL, functional exercise capacity and health care resource utilization. Compared to usual care, functional exercise capacity seemed to significantly improve after HBPR. While patient compliance with HBPR is good, two factors appeared to increase the ‘risk’ of non-compliance: expectations of patients to 1) complete daily diaries/activity logs and 2) engage in solely unsupervised exercise sessions.

**Conclusion:**

The overall quality for most outcomes was low to very low; however, HBPR seems to offer comparable short-term benefits to OPR.

**Supplementary Information:**

The online version contains supplementary material available at 10.1186/s12913-022-07779-9.

## Background

Chronic Obstructive Pulmonary Disease (COPD) is a progressive lung disease and a leading cause of morbidity and mortality worldwide with substantial economic and social burdens on individuals and society [1–3]. While COPD outpatient pulmonary rehabilitation (OPR) programs are well established, uptake remains suboptimal, in part because of difficulties in access to patients who do not live near to OPR [3, 4]. Home-based pulmonary rehabilitation program (HBPR) may represent an important strategy to improve patient access to this vital program. The purpose of this systematic review was to determine the effectiveness of HBPR programs for COPD patients.

## Methods

### Search strategy

A comprehensive, structured search strategy was developed iteratively by an experienced medical information specialist in consultation with the research team. It was peer-reviewed by another senior information specialist for quality assurance using the Peer Review of Electronic Search Strategies (PRESS) checklist (online supplementary appendix 1). The initial searches were conducted from March 1st to March 13th, 2020 and updated in November 17th 2021. They were also supplemented by manual searches of reference lists from included studies.

### Inclusion and exclusion criteria

Two reviewers independently screened the titles and abstracts of all citations. Full papers corresponding to potentially relevant citations were retrieved, divided among, and assessed by three reviewers for inclusion/exclusion according to criteria (Table 1). A pulmonary rehabilitation program was as defined by the American Thoracic Society and considered studies after 2009 in order to examine evidence reflecting current practice/guidelines [5]. Reviewers met to compare results and agree on the final set of studies to include. At both screening steps, consensus between reviewers was assessed using the Kappa statistics and found to be “substantial”.

**Table 1:** PICOS Elements of the effectiveness review

Parameter	Inclusion Criteria	Exclusion Criteria
Participants	• Patients with COPD	• Patients with Asthma • No patients (simulation studies)
Intervention	• Home-based pulmonary rehabilitation (home was defined as independent or supportive living environments)	• Pulmonary rehabilitation programs delivered in long-term care facilities or nursing homes • Not a program, as defined in the American Thoracic Society Consensus Statement • Program duration – less than 4 weeks
Comparator	• Outpatient pulmonary rehabilitation delivered in a hospital or community setting • Usual care (patients managed by their General Practitioner, specialist or both according to local practices)	• Inpatient pulmonary rehabilitation programs
Outcomes	• Safety • Health care resource utilization ○ Hospital admission ○ ER visits ○ Physician visits • Health Related Quality of Life (HRQoL) ○ Generic HRQoL tools such as EQ5D, SF36 or SF12 ○ Disease-specific HRQoL such as: ▪ COPD Assessment Test (CAT) ▪ Chronic Respiratory Disease Questionnaire (CRQ) ▪ St. George’s Respiratory Questionnaire (SGRQ) • Adherence • Frequency of exacerbation • Functional Exercise Capacity ○ Six-minute walk test/distance (6MWT/6MWD) ○ Incremental shuttle walk test (ISWT) ○ Endurance shuttle walk test (ESWT) • Maximal Exercise Capacity ○ Incremental cycle ergometry • Mental Health • Self-efficacy	• Studies without any defined clinical outcomes
*Study Design*	Comparative studies: • Randomized and non-randomized controlled trials (RCTs and non-RCTs) • Cohort studies • Case-control studies	• Non-English language • Expert reviews • Editorials and opinion pieces • Case-series • Studies published prior to 2009

### Data extraction and synthesis

Extracted data were tabulated to identify trends or patterns across studies and facilitate qualitative and quantitative comparative analyses. Key characteristics of included studies, their quality, potential sources of bias, and findings were synthesized narratively. A narrative synthesis of effectiveness outcomes across the studies was undertaken. Where studies appeared similar enough in patient populations, designs, and outcomes, meta-analyses were performed. Forest plots were used to display individual and pooled results. Pooled risk ratios for categorical data and mean differences with 95% confidence intervals (CIs) for continuous outcomes were calculated. A *p*-value < 0.05 was considered statistically significant. For each pooled estimate, the I^2^ statistic was calculated to measure the amount of heterogeneity across combined studies. Where the value exceeded 50% (indicating substantial heterogeneity), the pooled estimate was not used in the interpretation of the results. Publication bias was evaluated using funnel plots, where sufficient data were available from the meta-analyses [6]. Multiple publications from the same study were linked together in the tables.

### Assessment of study quality

RCTs were appraised using the Cochrane Collaboration ROB tool [7]. The methodological quality of the non-RCT interventional and comparative observational studies were evaluated using the Cochrane Risk of Bias Assessment Tool for Non-Randomized Studies (ACROBAT-NRSI) [8]. The quality of evidence relating to key outcomes of interest were assessed using the Grading of Recommendations, Assessment, Development and Evaluations (GRADE) tool [9]. GRADE assessment was based on the following outcomes: health-related quality of life (HRQoL), frequency of exacerbations, and COPD-related hospital admissions.

## Results

### Search results

**Fig. 1:**
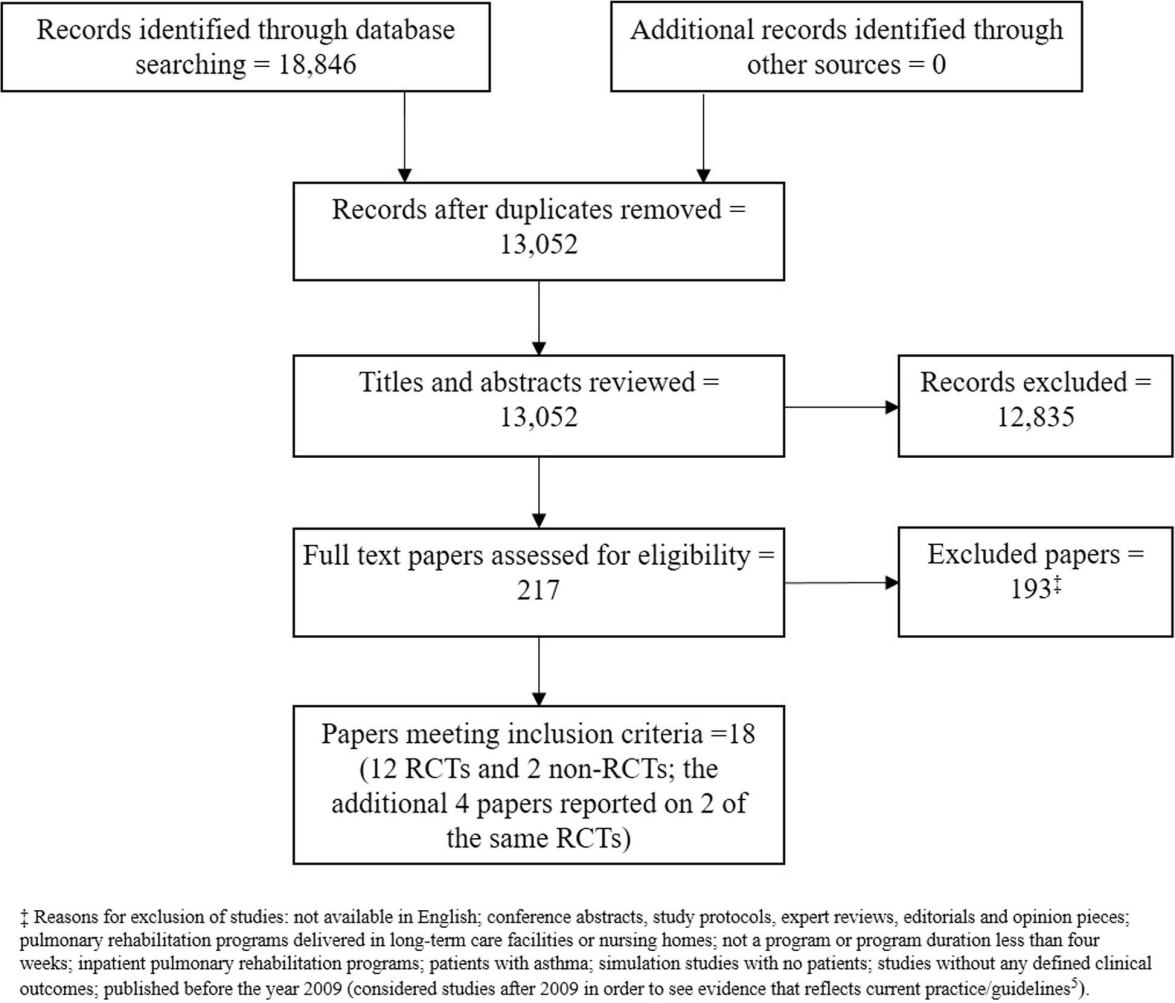
Preferred Reporting Items for Systematic Review and Meta-analysis (PRISMA) flow diagram for the systematic review and meta-analysis

A total of 18,846 citations were identified and screened, 217 were retrieved for full consideration, and 14 studies included - 12 RCTs [10–21] and 2 comparative observational [22, 23] studies (from 18 papers). The PRISMA diagram for the review is shown in Fig. 1 (additional information in online supplementary appendix 2).

### Characteristics of studies

Nine studies compared HBPR to ‘usual care’ [10, 11, 14–18, 20, 22], four to OPR [12, 13, 19, 21, 23], and one [19] compared HBPR to OPR or ‘usual care’. They were conducted between 2007 and 2017 in Australia (2 studies) [10, 12], Brazil (2) [16, 19], China (2) [14, 18], Iran (1) [15],Spain (2) [17, 22], the United States (1) [11], and the United Kingdom (4) [13, 20, 21, 23] (Table 2). Collectively, they included 2293 patients and all but four [10, 12, 13, 22] were conducted in single centers.

**Table 2:** Characteristics of included studies included in the systematic review

Study (country)	Study period (Design)	Number of centres	Number of participants	Follow-up	HBPR intervention supervision
HBPR vs ‘usual care’					
Lahham 2020 (Australia) [10]	Apr 2015- Nov 2017 (RCT)	Multiple centres	HBPR: 29 Usual care: 29	6 months	• Weekly phone calls with physiotherapist • Unsupervised home exercise training
Coultas 2018 (USA) [11]	Apr 2010- Apr 2014 (RCT)	Single centre	HBPR: 149 Usual care: 156	18 months	• Weekly telephone calls • Supervision not specified
Li 2018 (China) [14]	Jun 2014- Apr 2016 (RCT	Single centre	HBPR: 82 Usual care: 69	12 months	• Bi-weekly home visits for 2 months • Monthly home visit and weekly telephone calls for 4 months • Weekly telephone calls for 6 months • Unsupervised home exercise once per week (Supervised bi-weekly for first two months) • Unsupervised respiratory training three times per week
Khoshkesht 2015 (Iran) [15]	Dec 2010- Feb 2011 (RCT)	Single centre	HBPR: 35 Usual care: 35	7 weeks	• Weekly telephone calls with nurses Unsupervised home exercise training and breathing exercises
Pradella 2015 (Brazil) [16]	NR (RCT)	Single centre	HBPR: 32 Usual care: 18	8 weeks	• Weekly telephone call with nurse • Unsupervised exercise training
De Sousa Pinto 2014 (Spain) [17]	Oct 2009- Jun 2011 (RCT)	Single centre	HBPR: 29 Usual care: 21	12 weeks	• Weekly telephone calls • Supervised exercise twice per week for two weeks followed by twice per month • Unsupervised exercise weekly (frequency not specified)
Liu 2013 (China) [18]	Dec 2009- Oct 2011 (RCT)	Single centre	HBPR: 30 Usual care: 30	4 months	• Online program with system monitored program participation • Nurses contacted patients by telephone if they were not regularly logging into the system
Mendes de Oliveira 2010 (Brazil) [19]	Jan 2007- May 2009 (RCT)	Single centre	HBPR: 42 Usual care: 29	12 weeks	• Weekly telephone calls from health care provider • Home exercise program three times per week for 12 weeks (supervision not specified)
Moore 2009 (UK) [20]	NR (RCT)	Single centre	HBPR: 14 Usual care: 13	Mean ± SD HBPR: 8 ± 3 weeks Usual care: 7 ± 1 weeks	• Supervision not specified
Lalmolda 2017 (Spain) [22]	Jan 2011- NR Cohort study	Multiple centres	HBPR: 21 Usual care: 29	12 months	• Supervised program delivered by physiotherapist for one hour twice a week for 8 weeks
HBPR vs OPR					
Horton 2018 (UK) [21]	Nov 2007- Jul 2012 (RCT)	Single centre	HBPR: 145 OPR: 142	6 months	• Telephone calls during week two and week four • Unsupervised exercise program
Holland 2017 (Australia) [12]	Oct 2011- May 2015 (RCT)	Multiple centres	HBPR: 80 OPR: 86	12 months	• Weekly phone calls with physiotherapist • Unsupervised home exercise training
Mendes de Oliveira 2010 (Brazil) [19]	Jan 2007- May 2009 (RCT)	Single centre	HBPR: 42 OPR: 46	12 weeks	• Weekly telephone calls from health care provider • Home exercise program three times per week for twelve weeks (supervision not specified)
Nolan 2019 (UK) [23]	2012–2015 (Cohort study)	Single centre	HBPR: 154 OPR: 154	8 weeks	• Weekly telephone calls with physiotherapist • Unsupervised exercise training
Chaplin 2017 (UK) [13]	May 2013- Jul 2015 (RCT)	Multiple centres	HBPR: 51 OPR: 52	Mean ± SD HBPR: 11 ± 4 weeks OPR: NR	• Patients were contacted by a rehabilitation specialist weekly by email or telephone • Supervision not specified

Notes: No pulmonary rehabilitation (Usual care): patients were managed by their GP, specialist or both according to local practices

*HBPR* home-based pulmonary rehabilitation, *NR* not reported, *OPR* outpatient pulmonary rehabilitation, *RCT* randomized controlled trial, *SD* standard deviation

### HBPR program characteristics

All HBPR programs lasted at least 8 weeks. Except in three [11, 12, 18, 24–27] studies, programs began with one or more in-person training/education session(s) at an outpatient clinic or an initial in-home visit. The three [11, 12, 18, 24–27] exceptions delivered introductory sessions online or by video. Where described, sessions and in-home visits were conducted by nurses or physiotherapists. Additionally, all programs provided information booklets, manuals or workbooks and exercise prescription information, about which reported information varied. There were differences in specific components of and schedule for weekly in-home exercise, but most programs incorporated both strength and endurance training into activities to be performed unsupervised three times per week. However, four [12, 17, 22, 23] of the programs included supervised exercise. In the single [18] online HBPR, participation was monitored through the system itself, which collected log-on and log-off information. Patients logging on regularly were flagged and contacted by a nurse. Most programs asked patients to keep track of daily activities and symptoms in a diary or workbook. In one [15] case, patients used checklists. Programs employing special equipment or devices supplied them (hand and ankle weights [19], heart rate monitors [19], and pedometers [10, 12],). Weekly follow-up telephone calls by a nurse or physiotherapist to encourage or motivate patients and monitor progress were a part of almost all programs. Comparator interventions in studies were ‘usual care’ or OPR. ‘Usual care’, when described, varied significantly across studies, but typically comprised at least some form of in-person self-management/clinical needs assessment and advice on staying active and taking medications as prescribed, delivered through in-person education sessions and/or information booklets. OPR involved community-based supervised group sessions held two to three times weekly for seven to 12 weeks, and included exercise and education (online supplementary appendix 3, 4 and 5).

### Risk of bias

#### Results of risk of bias assessment

**Fig. 2:**
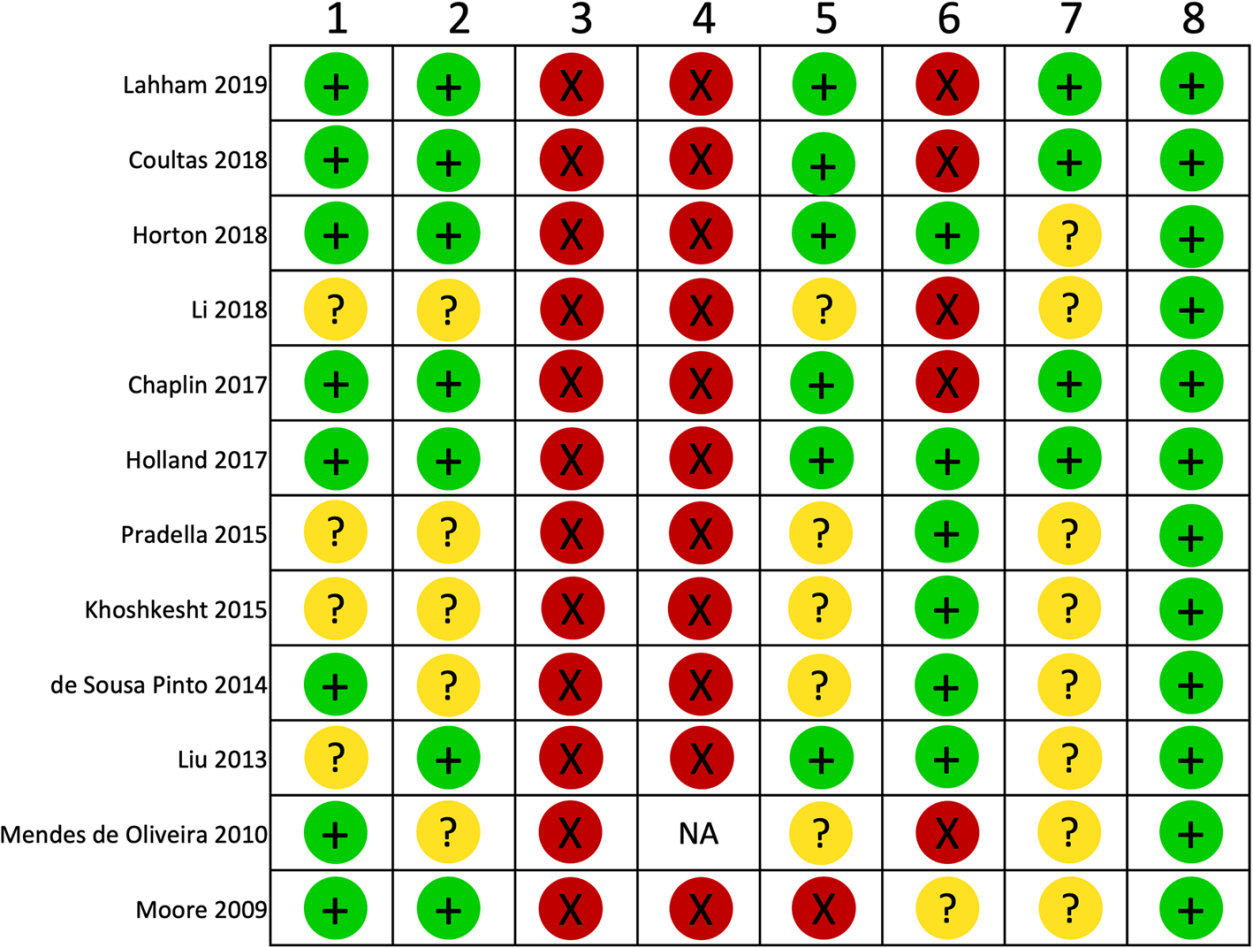
Cochrane risk of bias summary for included RCTs. (1) random sequence generation (selection bias); (2) allocation concealment (selection bias); (3) blinding of participants and personnel (performance bias); (4) blinding of outcome assessment (detection bias) (patient reported outcomes); (5) blinding of outcome assessment (detection bias) (other outcomes); (6) incomplete outcome data (attrition bias); (7) selective reporting (reporting bias); (8) other bias

The majority of RCTs provided a clear description of the objectives, interventions, outcomes and findings (online supplementary appendix 6). However, in two [15, 22] trials, ‘usual care’ was not defined. Four [10–13] of the RCTs had published or registered protocols pre-specifying outcomes, and in all five, such outcomes were the same as those reported in the trial results. In the remaining RCTs, it was not possible to determine if the results reflected all outcomes measured (Fig. 2).

**Fig. 3:**
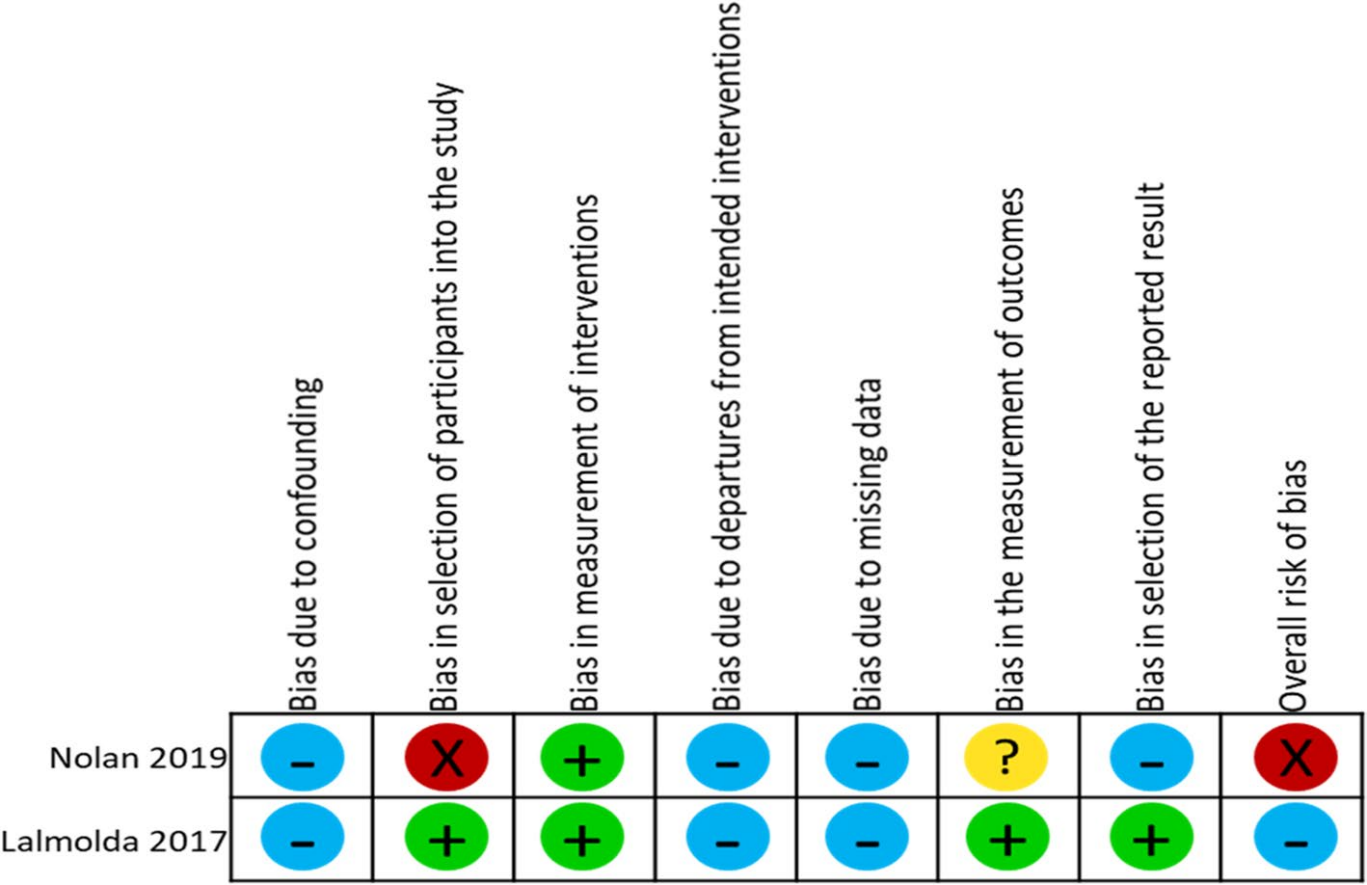
ACROBAT-NRSI summary

All non-randomized studies [22, 23] provided a clear description of the objectives, interventions, differences in patient characteristics and potential confounding variables between the HBPR and comparator groups, outcomes and findings (online supplementary appendix 7) (Fig. 3).

Of the 15 RCTs, eight [10–13, 17, 19–21] provided a clear description of the random sequence generation. In five [14–17, 19] RCTs, no details around allocation concealment were included. In the seven [10–13, 18, 20, 21] RCTs describing methods for ensuring blinding of patients and investigators, four [12, 18, 20, 21] used opaque sealed envelopes, two [11, 20] assigned the task to a researcher uninvolved in the trial, and two [10, 13] relied on web-based/online programs. In the two [10, 23] non-randomized studies, the risk of bias due to confounding was moderate, although known confounders were measured and controlled for through statistical analyses.

Six [10–13, 18, 21] RCTs reported that outcome assessors were blinded to the type of intervention. No information was provided in all but one [19] of the remaining trials. One [20] RCT explicitly stated that assessors were not blinded, rendering it at high risk for observer bias. None of the [10, 23] non-randomized studies mentioned blinding of assessors.

In five [10, 11, 13, 14, 19] RCTs, the risk of attrition bias was high but was low in six [12, 15–18, 21] RCTs, where missing data and reasons for withdrawals were similar between groups.

### Results from GRADE assessment

GRADE assessment was conducted on selected outcomes (Tables 3 and 4). The GRADE level was ‘very low’ or ‘low’ for all outcomes in HBPR vs. ‘usual care’ studies and 2 of 3 outcomes in HBPR vs OPR studies (Tables 3 and 4).

**Table 3:** Studies comparing HBPR to ‘usual care’

Outcomes	№ of participants (studies)	Certainty of the evidence (GRADE)	Relative effect (95% CI)	Anticipated absolute effects	
				Risk with ‘usual care’	Risk difference with HBPR
Health-related quality of life - COPD Assessment Test (CAT) scores following completion of intervention	151 (1 RCT)	⨁⨁◯◯ LOW^a,b^	–	Mean score = 0	**0** (0 to 0)
Frequency of exacerbations over duration of intervention	178 (2 RCTs)	⨁◯◯◯ VERY LOW^c,d,e^	not estimable	207 per 1000	207 fewer per 1000
Frequency of exacerbations over duration of intervention	48 (1 comparative observational study)	⨁◯◯◯ VERY LOW^e^	not estimable	276 per 1000	276 fewer per 1000
6 min walk test (6MWT/6MWD) in meters at the end of PR	745 (7 RCTs)	⨁◯◯◯ VERY LOW^d,f,g^	–	not pooled	not pooled
Hospital admissions rate related to COPD at the end of PR	305 (1 RCT)	⨁⨁◯◯ LOW^a,e^	not estimable	301 per 1000	301 fewer per 1000
Hospital admissions rate related to COPD at the end of PR	48 (1 comparative observational study)	⨁◯◯◯ VERY LOW^b^	not estimable	138 per 1000	138 fewer per 1000
Health-related quality of life - St. George’s respiratory questionnaire (SGRQ) total score following completion of intervention	160 (3 RCTs)	⨁⨁◯◯ LOW^b,h^	–	not pooled	not pooled

The risk in the intervention group is based on the assumed risk in the comparison group and the relative effect of the intervention

Explanations

^a^Study at high risk of attrition bias

^b^Small sample size

^c^One study at high risk of attrition bias and one study at high risk of detection bias

^d^Point estimates are different across studies

^e^Lower number of events

^f^Studies at high risk of attrition bias

^g^Indirect outcome

^h^Studies at high risk of detection bias

**Table 4:** Studies comparing HBPR to OPR

Outcomes	№ of participants (studies)	Certainty of the evidence (GRADE)	Relative effect (95% CI)	Anticipated absolute effects	
				Risk with OPR	Risk difference with HBPR
Health-related quality of life - COPD Assessment Test (CAT) scores following completion of intervention	103 (1 RCT)	⨁⨁◯◯ LOW^a,b^	–	not pooled	not pooled
Frequency of exacerbations over duration of intervention	NR	NR	NR	NR	NR
6 min walk test (6MWT/6MWD) in meters at the end of PR	254 (2 RCTs)	⨁◯◯◯ VERY LOW^b,c,d^	–	not pooled	not pooled
Hospital admissions rate related to COPD at the end of PR	287 (1 RCT)	⨁⨁⨁◯ MODERATE^b^	not estimable	not pooled	not pooled
Health-related quality of life - St. George’s respiratory questionnaire (SGRQ) total score following completion of intervention	NR	NR	NR	NR	NR

Explanations

^a^Study at high risk of performance, detection and attrition bias

^b^Small sample size

^c^One study at high risk of attrition bias

^d^Indirect outcome

### Summary results of effectiveness

#### Safety

Three [11, 12, 21] studies reported adverse event rates, but none specified the type of adverse event. One [11] study comparing HBPR to usual care, and two [12, 21] studies comparing HBPR to OPR showed no statistically significant differences between groups (online supplementary appendix 8).

#### Health care resource utilization (hospital admissions, ER visits, and physician visits)

##### HBPR compared to ‘usual care’

In one of two [11, 22, 24, 25] studies comparing the percentage of patients with one or more COPD-related hospital admissions over a 12 to 18 month period, there were no statistically significant differences between groups. In the second [11, 24, 25] study, the percentage of patients with admission in the HBPR group was almost half that of the ‘usual care’ group (19% versus 30%; statistical significance of the difference not reported).

##### HBPR compared to OPR

One [12] study concluded that the impact of HBPR and OPR on health services utilization was similar.

#### Health-related quality of life (HRQoL)

Different disease-specific instruments were used, including the COPD assessment test (CAT), chronic respiratory disease questionnaire (CRQ), and the St. George’s respiratory questionnaire (SGRQ). Measurements were also at different points in time as the duration of rehabilitation programs varied among studies (online supplementary appendix 9, 10 and 11).

##### HBPR compared to usual care

In the study [14] using the CAT, short-term improvements in scores were statistically significantly greater in the HBPR group, but both groups experienced clinically meaningful improvements (a change in scores of at least 2 points [28]). However, 6 months after completion of HBPR, improvements from baseline were similar between groups. Two studies [10, 11, 24, 25] using CRQ found no statistically significant differences in changes between treatment groups on any of the 4 CRQ domains from baseline to several months after HBPR or ‘usual care’. However, in one [10], both groups reported clinically meaningful improvements in the dyspnea, fatigue and mastery domains at 2 and 6 months of follow-up (a change in score of at least 0.5 points). In the other [11, 24, 25], neither group experienced clinically meaningful improvements in the dyspnea domains. In contrast, the single study [20] that measured HRQoL directly following completion of HBPR reported statistically significant improvements in dyspnea, emotional function and fatigue among patients who had HBPR, but not among those who received ‘usual care’. In two [16, 17] of the three [16–18] studies using the SGRQ there were statistically significantly greater improvements in total scores (from baseline to end of treatment) with HBPR. However, for individual domains, scores varied between studies; in one study, changes in all domain scores were similar between groups and in the other, those for the ‘activity’ and ‘impact’ domains were greater among HBPR patients. The third [18] study assessed HRQoL 10 months post HBPR or ‘usual care’. There were no statistically significant differences between groups, except for social functioning and psychological disturbances resulting from COPD (i.e., ‘impact’ domain), which improved with HBPR and worsened with ‘usual care’.

##### HBPR compared to OPR

**Fig. 4:**
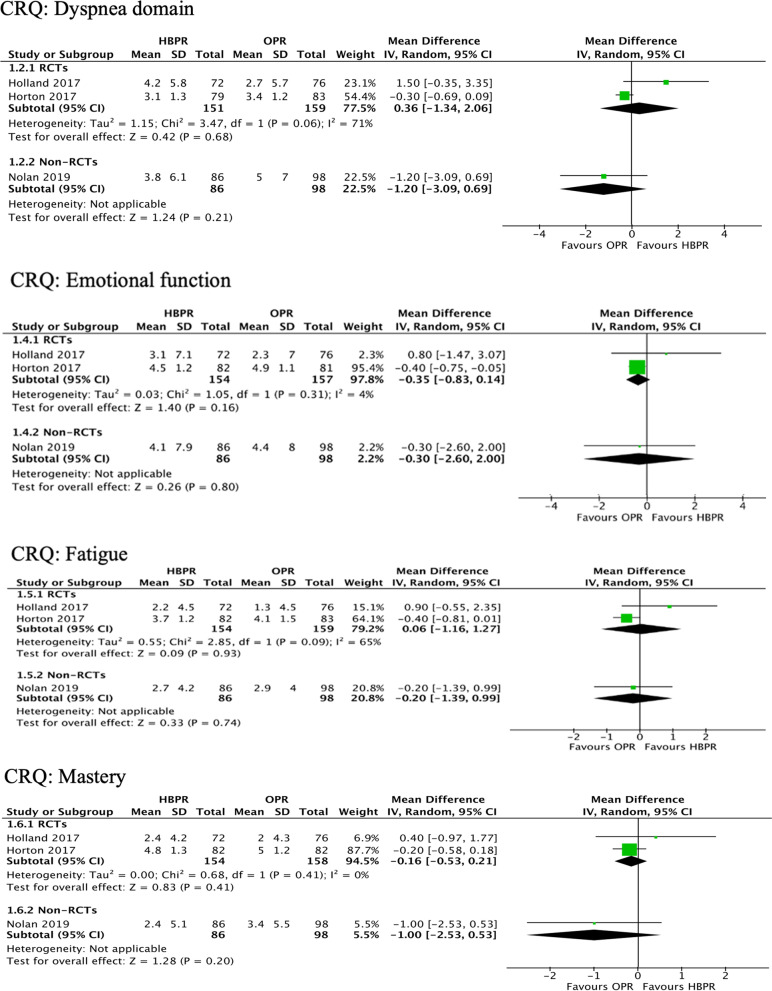
Mean differences in health-related quality of life after completion of 2-month active pulmonary rehabilitation phase in studies comparing HBPR with OPR

In one [13] study using CAT before and after completion of the programs, actual scores were not presented, but the reported *p*-value was not statistically significant (Fig. 4). Three [12, 21, 23] studies used CRQ and measured HRQoL at program completion, and meta-analyses were possible in 4 domains of the instrument: dyspnea, emotional, fatigue and mastery. Two [15, 26] found no statistically significant differences between groups in these domains after 2 months of pulmonary rehabilitation. By contrast, in the third [21] study, scores were statistically significantly lower in the emotional, fatigue and mastery domains among HBPR patients. However, 4 months after the end of the program, no statistically significant differences were reported.

#### Adherence to/compliance with treatment

##### HBPR compared to usual care

With one exception [17], at least 79% of patients completed HBPR [10, 11, 15, 16, 18–20, 22, 24, 25] Of the three [17, 19, 20] studies reporting lower rates of adherence with HBPR, ‘usual care’ did not include an exercise component or diary for patients to log symptoms and/or activities on a daily basis.

##### HBPR compared to OPR

The percentage of patients completing HBPR was higher in three studies [12, 19, 21] but lower in two [13, 23], but the statistical significance was not reported. In one study [13], with lower adherence to HBPR, patients withdrew because it was too time-consuming or preferred a classroom setting or had computer problems affecting access (online supplementary appendix 12).

#### Frequency of exacerbations

##### HBPR compared ‘usual care’

Two studies [14, 22] showed no statistically significant differences in exacerbation rates during the rehabilitation phase between groups (online supplementary appendix 13).

#### Functional exercise capacity and activity levels

Different assessment tools were used to measure functional exercise capacity; the most commonly applied measure, the six-minute walk test. Others were the incremental shuttle walk test (ISWT) and the endurance shuttle walk test (ESWT).

##### HBPR compared to usual care

Across studies [11, 14, 18, 19, 22, 24, 25], the distance walked in 6 min statistically significantly increased among patients who received HBPR, indicating an improvement in exercise capacity. In some studies, it also increased among patients who received ‘usual care’, but the amount of increase was statistically significantly less than that reported for HBPR.

Five [10, 11, 14, 17, 19, 24, 25] out of six studies demonstrated that patients receiving HBPR had clinically meaningful improvements by the end of follow-up (change in distance of at least 30 m). However, one study [11, 24, 25] reported that at 18 months, only patients with serious COPD showed meaningful improvements in this test.

##### HBPR compared to OPR

In all four studies [13, 19, 21, 23], the distance walked in 6 min statistically significantly increased after HBPR and OPR, and the gains were similar between programs. However, one study [21] that measured ISWT reported no clinically meaningful improvements (change in distance of at least 48 m [29]) after HBPR or OPR.

#### Mental health

Studies used the Beck depression inventory, and the Hospital Anxiety and Depression scale (HADS) to assess changes in depression and anxiety (online supplementary appendix 14).

##### HBPR compared to ‘usual care’

In one [14] study, statistically significant improvements in scores on the Beck Depression Inventory were observed in both groups, but no statistical estimates of between-group measures were provided.

##### HBPR compared to OPR

Two [13, 21] of the five studies used the HADS. Within groups, post-program scores were similar to baseline, indicating that neither form of pulmonary rehabilitation reduced anxiety and depression among patients.

#### Self-efficacy

##### HBPR compared to ‘usual care’

One [15] study assessed self-efficacy changes between baseline and 2 months (the length of the active rehabilitation). HBPR patients had statistically significantly greater confidence in their ability to manage or avoid dyspnea during events across all five domains of the questionnaire compared to ‘usual care’.

##### HBPR compared to OPR

Three [12, 13, 21] studies used the PRAISE (Pulmonary Rehabilitation Adapted Index of Self-efficacy) tool [30]. However, only two [13, 21] reported statistical estimates of data variability between groups when administered to patients before and after the active rehabilitation phase (3 months in one study and 2 months in the other). Within and between groups, pre and post-treatment scores were similar, indicating that the confidence level among patients to self-manage their disease did not increase with HBPR or OPR (online supplementary appendix 15).

## Discussion

This review examined the effectiveness of HBPR compared to usual care or OPR for safety, patient compliance, HRQoL, exercise capacity, self-efficacy, mental health, and health care resource utilization. Overall, HBPR appears to be comparable to standard OPR in terms of safety, HRQoL and exercise capacity. While there is considerable evidence supporting the effectiveness HBPR for COPD patients, study quality is low, and therefore findings should be interpreted with caution.

Although there have been previous reviews of HBPR published [31, 32], the current review included a larger number of primary studies and outcome measures. Only two common outcome measures were reported in all 3 of these reviews – HRQoL and exercise capacity, based on the CRQ and 6 min walk test, respectively. With respect to these two outcomes, Chen et al. also found that improvements after HBPR were similar to OPR [32]. By contrast, the earlier Liu et al. study reported improved HRQoL and functional capacity in the HBPR groups. However, it included patients in the control groups undergoing various types of interventions (including patients who did not have any form of exercise) [31]. In the present review, additional system outcomes, including measures of mental health and health resource utilization, have been included for the first time.

As HBPR takes place in the home with limited supervision, there could be a concern for patient safety. However, in the limited number of studies that reported on safety, importantly, there were no significant differences in reported adverse events between HBPR and OPR. This is consistent with a recent Cochrane review that did not identify any safety issues with telerehabilitation in patients with chronic respiratory diseases [33]. Combined, these results suggest that HBPR is safe and poses no greater risk than standard OPR.

Adherence to PR is key to achieve improvements in health outcomes and to facilitate behaviour change. HBPR has the advantage over traditional in-person PR in that barriers such as daily commute and weather should not affect adherence. Home-based programs could also be provided to rural and remote areas without space and workforce resources for a OPR. Further, the COVID-19 pandemic has forced heath systems to introduce home-based programs, including pulmonary rehabilitation programs. As a result, it has become evident that there might be additional groups of COPD patients who might benefit from HBPR. However, the risk of non-compliance to HBPR in the studies reviewed was associated with two PR program factors: 1) if there are expectations of patients to complete daily diaries/activity logs, or 2) if the program engaged in solely unsupervised exercise sessions. Requiring patients to fill out daily logs could become burdensome and represent an additional barrier to participation. Technological advances, e.g., remote-wireless activity monitors, may reduce this challenge in the future. Supervision/feedback during the exercise sessions would provide social interaction and positive reinforcement opportunities, which are key mechanisms for enhancing self-efficacy [34]. Self-efficacy has been shown to predict attendance at PR [35] and long-term adherence to exercise programs [36]. Further, supervision would increase the likelihood that the patient is following all components of their exercise program (e.g. target exercise intensity), which would maximize benefits from PR and the likelihood that patients would perceive such benefits. Patients have reported that expectations of benefits are critical for their engagement in PR and exercise programs [37–40].

The inclusion of an individualized exercise plan appeared to facilitate greater improvements in exercise capacity with HBPR. An individualized exercise plan would require a thorough patient assessment, including evaluation of exercise capacity, at the start of rehab, as well as an exercise program be developed based on fundamental exercise principles. These are entirely consistent with recently published quality indicators for PR [41]. Similar to the supervision component discussed previously, an individualized exercise plan will facilitate an exercise program with the appropriate aerobic stimulus/training load. Aerobic training load has been shown to be an important determinant in improvement in exercise tolerance with PR [42]. Combined, these findings support the need for individualized exercise programs in order to maximize health outcomes in patients participating in HBPR.

This review demonstrates that neither HBPR nor OPR appear to reduce health care resource utilization. Across the studies comparing HBPR to ‘usual care,’ the findings varied, but there were no statistically significant differences between groups, except in terms of the 6 min walk test. The one study that assessed the statistical significance of differences in hospital admissions suggests that the impact of HBPR and OPR on health services utilization was similar.

### Limitations

This systematic review has several limitations. First, there is the possible risk of bias due to missing information in the included studies. Furthermore, included studies provided limited descriptions of the study randomization process, and the studies varied in components of the interventions. Second, the study was restricted to English language studies, which might have led to the exclusion of possibly relevant studies. The review was also limited to work published in 2009 or later, and therefore some papers published before that time [43] were excluded. Also, it was not possible to perform a meta-analysis on most outcomes due to a high level of heterogeneity and limited data.

## Conclusion

In conclusion, HBPR is an alternative approach which appears as safe as OPR, and HBPR outcomes were similar to standard pulmonary rehabilitation programs. Although there is a considerable amount of evidence relating to these programs’ effectiveness for COPD patients, its quality is low and should be interpreted with caution.

## Supplementary Information

**Additional file 1: Supplementary Appendix 1.** Literature search results. **Supplementary Appendix 2.** Summary of included studies of home-based pulmonary rehabilitation. **Supplementary Appendix 3.** Characteristics of patient populations across included studies. **Supplementary Appendix 4.** Summary of HBPR comparator interventions in included studies. **Supplementary Appendix 5.** Home based pulmonary rehabilitation program components. **Supplementary Appendix 6.** Risk of bias in RCTs. **Supplementary Appendix 7.** Risk of bias in non-randomized studies. **Supplementary Appendix 8.** Adverse events and deaths during follow-up period. **Supplementary Appendix 9.** Health-related quality of life –CAT, AQ 20, or VSRQ. **Supplementary Appendix 10.** Health related quality of life - Chronic Respiratory Disease Questionnaire (CRQ). **Supplementary Appendix 11.** Health related quality of life – St George’s Respiratory Questionnaire. **Supplementary Appendix 12.** Patient adherence to/compliance with HBPR or comparator. **Supplementary Appendix 13.** Frequency of exacerbations, hospital admissions and ER visits. **Supplementary Appendix 14.** Mental health. **Supplementary Appendix 15.** Self-efficacy.

## Data Availability

All data relevant to the study are included in the article or uploaded as supplementary information.
