# Supplementary material for: Effectiveness of home-based pulmonary rehabilitation programs for patients with chronic obstructive pulmonary disease (COPD): systematic review
Source: BMC Health Serv Res. 2022 Apr 26;22:557. doi: 10.1186/s12913-022-07779-9 (PMC9039605; doi:10.1186/s12913-022-07779-9)
Supplement: Supplementary file 1 — Additional file 1: Supplementary Appendix 1. Literature search results. Supplementary Appendix 2. Summary of included studies of home-based pulmonary rehabilitation. Supplementary Appendix 3. Characteristics of patient populations across included studies. Supplementary Appendix 4. Summary of HBPR comparator interventions in included studies. Supplementary Appendix 5. Home based pulmonary rehabilitation program components. Supplementary Appendix 6. Risk of bias in RCTs. Supplementary Appendix 7. Risk of bias in non-randomized studies. Supplementary Appendix 8. Adverse events and deaths during follow-up period. Supplementary Appendix 9. Health-related quality of life –CAT, AQ 20, or VSRQ. Supplementary Appendix 10. Health related quality of life - Chronic Respiratory Disease Questionnaire (CRQ). Supplementary Appendix 11. Health related quality of life – St George’s Respiratory Questionnaire. Supplementary Appendix 12. Patient adherence to/compliance with HBPR or comparator. Supplementary Appendix 13. Frequency of exacerbations, hospital admissions and ER visits. Supplementary Appendix 14. Mental health. Supplementary Appendix 15. Self-efficacy. [file 12913_2022_7779_MOESM1_ESM.docx]

# Supplemental materials

[Supplementary Appendix 1: Literature search results 2](#_Toc89180456)

[Supplementary Appendix 2. Summary of included studies of home-based pulmonary rehabilitation 16](#_Toc89180457)

[Supplementary Appendix 3. Characteristics of patient populations across included studies 22](#_Toc89180458)

[Supplementary Appendix 4. Summary of HBPR comparator interventions in included studies 29](#_Toc89180459)

[Supplementary Appendix 5. Home based pulmonary rehabilitation program components 36](#_Toc89180460)

[Supplementary Appendix 6: Risk of bias in RCTs 53](#_Toc89180461)

[Supplementary Appendix 7: Risk of bias in non-randomized studies 60](#_Toc89180462)

[Supplementary Appendix 8. Adverse events and deaths during follow-up period 61](#_Toc89180463)

[Supplementary Appendix 9. Health-related quality of life –CAT, AQ 20, or VSRQ 64](#_Toc89180464)

[Supplementary Appendix 10. Health related quality of life - Chronic Respiratory Disease Questionnaire (CRQ) 67](#_Toc89180465)

[Supplementary Appendix 11. Health related quality of life – St George’s Respiratory Questionnaire 70](#_Toc89180466)

[Supplementary Appendix 12. Patient adherence to/compliance with HBPR or comparator 73](#_Toc89180467)

[Supplementary Appendix 13. Frequency of exacerbations, hospital admissions and ER visits 77](#_Toc89180468)

[Supplementary Appendix 14. Mental health 80](#_Toc89180469)

[Supplementary Appendix 15. Self-efficacy 82](#_Toc89180470)

Supplementary Appendix 1: Literature search results

Initial search: 2020 Mar 11

Updated search: 2021 Nov 17

**Ovid Multifile**

Database: Ovid MEDLINE(R) ALL <1946 to November 16, 2021>, Embase <1974 to 2021 November 16>

Search Strategy:

--------------------------------------------------------------------------------

1 Lung Diseases, Obstructive/ (18430)

2 exp Pulmonary Disease, Chronic Obstructive/ (210576)

3 exp Emphysema/ or exp Pulmonary Emphysema/ (77378)

4 ((chronic adj2 obstructi*) and (pulmonary or airway* or air way* or lung$1 or airflow* or air flow*)).tw,kf. (151688)

5 (COPD or COAD).tw,kf. (152304)

6 (chronic adj2 bronchitis).tw,kf. (24878)

7 emphysema*.tw,kf. (66128)

8 exp Respiratory Therapy/ (129631)

9 respirat* therap*.tw,kf. (8298)

10 ((pulmonary or respirat*) adj3 rehab*).tw,kf. (14240)

11 or/1-10 [COPD, Incl. RESPIRATORY THERAPY/REHAB] (493893)

12 Home Care Services/ (89105)

13 Home Care Services, Hospital-Based/ (66638)

14 Home Health Nursing/ (62822)

15 Home Nursing/ (71093)

16 ((domicil* or home?) adj3 (care or health care or healthcare)).tw,kf. (86795)

17 ((domicil* or home?) adj3 (assist* or manag* or monitor* or network* or program* or rehab* or resourc* or servic* or support*)).tw,kf. (78418)

18 ((domicil* or home?) adj3 model*).tw,kf. (5050)

19 homecare.tw,kf. (3447)

20 (home-based or homebased or domicile-based).tw,kf. (29771)

21 ((domicil* or home?) adj3 dwelling).tw,kf. (1908)

22 ((domicil* or home?) adj3 environment*).tw,kf. (20140)

23 ((domicil* or home?) adj3 session?).tw,kf. (2117)

24 ((domicil* or home?) adj3 setting?).tw,kf. (16855)

25 home nurs*.tw,kf. (3982)

26 "at home".tw,kf. (136108)

27 "in home".tw,kf. (27909)

28 (patient* home? or patient* domicil*).tw,kf. (8413)

29 (home? adj2 domicil*).tw,kf. (134)

30 (home* adj2 PR).tw,kf. (281)

31 HBPR.tw,kf. (71)

32 home?.ti. (184416)

33 Self Management/ (59074)

34 Self Care/ (100704)

35 limit 34 to yr="2015-2017" (17726)

36 ((care or manag*) adj2 self).tw,kf. (110240)

37 ((care or manag*) adj2 himself).tw,kf. (186)

38 ((care or manag*) adj2 herself).tw,kf. (188)

39 ((care or manag*) adj2 themsel*).tw,kf. (3265)

40 or/12-33,35-39 [HOME-BASED SERVICES] (556001)

41 11 and 40 [HOME-BASED PR] (14869)

42 exp Animals/ not Humans/ (17185131)

43 41 not 42 [ANIMAL-ONLY REMOVED] (11858)

44 (comment or editorial or news or newspaper article).pt. (2258899)

45 (letter not (letter and randomized controlled trial)).pt. (2351413)

46 43 not (44 or 45) [OPINION PIECES REMOVED] (11267)

47 46 use medall [MEDLINE RECORDS] (6273)

48 (2020031* or 2020032* or 2020033* or 202004* or 202005* or 202006* or 202007* or 202008* or 202009* or 202010* or 202011* or 202012* or 2021*).dt. (2614587)

49 47 and 48 [MEDLINE RECORDS - UPDATE PERIOD] (679)

50 obstructive airway disease/ (2195)

51 chronic obstructive lung disease/ (194257)

52 emphysema/ or exp lung emphysema/ (46446)

53 ((chronic adj2 obstructi*) and (pulmonary or airway* or air way* or lung$1 or airflow* or air flow*)).tw,kw. (147007)

54 (COPD or COAD).tw,kw. (151415)

55 (chronic adj2 bronchitis).tw,kw. (24351)

56 emphysema*.tw,kw. (64893)

57 exp respiratory care/ (4682)

58 respirat* therap*.tw,kf. (8298)

59 pulmonary rehabilitation/ (7782)

60 ((pulmonary or respirat*) adj3 rehab*).tw,kf. (14240)

61 or/50-60 [COPD, Incl. RESPIRATORY THERAPY/REHAB] (356859)

62 home care/ (99781)

63 home monitoring/ (5040)

64 home oxygen therapy/ (1268)

65 home rehabilitation/ (858)

66 home respiratory care/ (104)

67 visiting nursing service/ (206)

68 ((domicil* or home?) adj3 (care or health care or healthcare)).tw,kw. (84230)

69 ((domicil* or home?) adj3 (assist* or manag* or monitor* or network* or program* or rehab* or resourc* or servic* or support*)).tw,kw. (76734)

70 ((domicil* or home?) adj3 model*).tw,kw. (5039)

71 homecare.tw,kw. (3410)

72 (home-based or homebased or domicile-based).tw,kw. (29424)

73 ((domicil* or home?) adj3 environment*).tw,kw. (19992)

74 ((domicil* or home?) adj3 session?).tw,kw. (2116)

75 ((domicil* or home?) adj3 setting?).tw,kw. (16820)

76 home nurs*.tw,kw. (3970)

77 "at home".tw,kw. (135924)

78 "in home".tw,kw. (27862)

79 (patient* home? or patient* domicil*).tw,kw. (9110)

80 (home? adj2 domicil*).tw,kw. (255)

81 (home* adj2 PR).tw,kw. (283)

82 HBPR.tw,kw. (71)

83 home?.ti. (184416)

84 self care/ (100704)

85 ((care or manag*) adj2 self).tw,kw. (106696)

86 ((care or manag*) adj2 himself).tw,kw. (186)

87 ((care or manag*) adj2 herself).tw,kw. (187)

88 ((care or manag*) adj2 themsel*).tw,kw. (3265)

89 or/62-88 [HOME-BASED SERVICES] (566948)

90 61 and 89 [HOME-BASED PR] (12573)

91 exp animal/ or exp animal experimentation/ or exp animal model/ or exp animal experiment/ or nonhuman/ or exp vertebrate/ (54559693)

92 exp human/ or exp human experimentation/ or exp human experiment/ (42851254)

93 91 not 92 (11710185)

94 90 not 93 [ANIMAL-ONLY REMOVED] (12537)

95 (editorial or letter).pt. (3652024)

96 94 not 95 [OPINION PIECES REMOVED] (12061)

97 94 use oemezd [EMBASE RECORDS] (8598)

98 (2020031* or 2020032* or 2020033* or 202004* or 202005* or 202006* or 202007* or 202008* or 202009* or 202010* or 202011* or 202012* or 2021*).dc. (3577668)

99 97 and 98 [EMBASE RECORDS - UPDATE PERIOD] (1399)

100 49 or 99 [BOTH DATABASES] (2078)

101 limit 100 to english (2011)

102 remove duplicates from 101 (1542) [TOTAL UNIQUE RECORDS]

103 102 use medall [MEDLINE RECORDS] (630)

104 102 use oemezd [EMBASE RECORDS] (912)

***************************

**Cochrane/EBM**

Database: EBM Reviews - Cochrane Central Register of Controlled Trials <October 2021>, EBM Reviews - Cochrane Database of Systematic Reviews <2005 to November 11, 2021>

Search Strategy:

--------------------------------------------------------------------------------

1 Lung Diseases, Obstructive/ (3080)

2 exp Pulmonary Disease, Chronic Obstructive/ (6063)

3 exp Emphysema/ or exp Pulmonary Emphysema/ (474)

4 ((chronic adj2 obstructi*) and (pulmonary or airway* or air way* or lung$1 or airflow* or air flow*)).ti,ab,kw. (16228)

5 (COPD or COAD).ti,ab,kw. (17990)

6 (chronic adj2 bronchitis).ti,ab,kw. (1945)

7 emphysema*.ti,ab,kw. (1529)

8 exp Respiratory Therapy/ (8366)

9 respirat* therap*.ti,ab,kw. (547)

10 ((pulmonary or respirat*) adj3 rehab*).ti,ab,kw. (2668)

11 or/1-10 [COPD, Incl. RESPIRATORY THERAPY/REHAB] (34414)

12 Home Care Services/ (1889)

13 Home Care Services, Hospital-Based/ (238)

14 Home Health Nursing/ (8)

15 Home Nursing/ (279)

16 ((domicil* or home?) adj3 (care or health care or healthcare)).ti,ab,kw. (6922)

17 ((domicil* or home?) adj3 (assist* or manag* or monitor* or network* or program* or rehab* or resourc* or servic* or support*)).ti,ab,kw. (12174)

18 ((domicil* or home?) adj3 model*).ti,ab,kw. (495)

19 homecare.ti,ab,kw. (186)

20 (home-based or homebased or domicile-based).ti,ab,kw. (7869)

21 ((domicil* or home?) adj3 dwelling).ti,ab,kw. (261)

22 ((domicil* or home?) adj3 environment*).ti,ab,kw. (1729)

23 ((domicil* or home?) adj3 session?).ti,ab,kw. (1656)

24 ((domicil* or home?) adj3 setting?).ti,ab,kw. (1818)

25 home nurs*.ti,ab,kw. (389)

26 "at home".ti,ab,kw. (47529)

27 "in home".ti,ab,kw. (47446)

28 (patient* home? or patient* domicil*).ti,ab,kw. (1717)

29 (home? adj2 domicil*).ti,ab,kw. (19)

30 (home* adj2 PR).ti,ab,kw. (58)

31 HBPR.ti,ab,kw. (9)

32 home?.ti. (13184)

33 Self Management/ (587)

34 Self Care/ (4270)

35 limit 34 to yr="2015-2017" (908)

36 ((care or manag*) adj2 self).ti,ab,kw. (16154)

37 ((care or manag*) adj2 himself).ti,ab,kw. (12)

38 ((care or manag*) adj2 herself).ti,ab,kw. (11)

39 ((care or manag*) adj2 themsel*).ti,ab,kw. (188)

40 or/12-33,35-39 [HOME-BASED SERVICES] (63907)

41 11 and 40 [HOME-BASED PR] (2553)

42 (202003* or 202004* or 202005* or 202006* or 202007* or 202008* or 202009* or 202010* or 202011* or 202012* or 2021*).up. (967969)

43 41 and 42 [UPDATE PERIOD] (1382)

44 remove duplicates from 43 (1370) [TOTAL UNIQUE RECORDS]

45 44 use cctr [CENTRAL RECORDS – UPDATE PERIOD] (1354)

46 44 use coch [CDSR RECORDS – UPDATE PERIOD] (16)

***************************

**CINAHL**

| # | Query | Limiters/Expanders | Results |
| --- | --- | --- | --- |
| S46 | S44 AND S45 | Expanders - Apply equivalent subjects  Search modes - Find all my search terms | 344 |
| S45 | EM 20200301-20211231 | Expanders - Apply equivalent subjects  Search modes - Find all my search terms | 636,487 |
| S44 | S13 AND S41 | Limiters - Exclude MEDLINE records  Expanders - Apply equivalent subjects  Search modes - Find all my search terms | 2,393 |
| S43 | S13 AND S41 | Expanders - Apply equivalent subjects  Search modes - Find all my search terms | 4,892 |
| S42 | S13 AND S41 | Expanders - Apply equivalent subjects  Search modes - Find all my search terms | 4,892 |
| S41 | S14 OR S15 OR S16 OR S17 OR S18 OR S19 OR S20 OR S21 OR S22 OR S23 OR S24 OR S25 OR S26 OR S27 OR S28 OR S29 OR S30 OR S31 OR S32 OR S33 OR S34 OR S35 OR S36 OR S37 OR S38 OR S39 OR S40 | Expanders - Apply equivalent subjects  Search modes - Find all my search terms | 224,918 |
| S40 | TI ( (care or manag*) N2 themsel* ) OR AB ( (care or manag*) N2 themsel* ) | Expanders - Apply equivalent subjects  Search modes - Find all my search terms | 1,541 |
| S39 | TI ( (care or manag*) N2 herself ) OR AB ( (care or manag*) N2 herself ) | Expanders - Apply equivalent subjects  Search modes - Find all my search terms | 86 |
| S38 | TI ( (care or manag*) N2 himself ) OR AB ( (care or manag*) N2 himself ) | Expanders - Apply equivalent subjects  Search modes - Find all my search terms | 54 |
| S37 | TI ( (care or manag*) N2 self ) OR AB ( (care or manag*) N2 self ) | Expanders - Apply equivalent subjects  Search modes - Find all my search terms | 37,049 |
| S36 | (MH "Self Care") | Expanders - Apply equivalent subjects  Search modes - Find all my search terms | 42,721 |
| S35 | (MH "Self-Management") | Expanders - Apply equivalent subjects  Search modes - Find all my search terms | 1,684 |
| S34 | TI home# | Expanders - Apply equivalent subjects  Search modes - Find all my search terms | 69,247 |
| S33 | TI HBPR OR AB HBPR | Expanders - Apply equivalent subjects  Search modes - Find all my search terms | 2 |
| S32 | TI home# N2 PR OR AB home# N2 PR | Expanders - Apply equivalent subjects  Search modes - Find all my search terms | 32 |
| S31 | TI home# N2 domicil* OR AB home# N2 domicil* | Expanders - Apply equivalent subjects  Search modes - Find all my search terms | 50 |
| S30 | TI ( (patient* N0 home#) or (patient* N0 domicil*) ) OR AB ( (patient* N0 home#) or (patient* N0 domicil*) ) | Expanders - Apply equivalent subjects  Search modes - Find all my search terms | 2,748 |
| S29 | TI "in home" OR AB "in home" | Expanders - Apply equivalent subjects  Search modes - Find all my search terms | 142,911 |
| S28 | TI "at home" OR AB "at home" | Expanders - Apply equivalent subjects  Search modes - Find all my search terms | 142,911 |
| S27 | TI home N0 nurs* OR AB home N0 nurs* | Expanders - Apply equivalent subjects  Search modes - Find all my search terms | 25,423 |
| S26 | TI ( (domicil* or home#) N3 setting# ) OR AB ( (domicil* or home#) N3 setting# ) | Expanders - Apply equivalent subjects  Search modes - Find all my search terms | 6,303 |
| S25 | TI ( (domicil* or home#) N3 session# ) OR AB ( (domicil* or home#) N3 session# ) | Expanders - Apply equivalent subjects  Search modes - Find all my search terms | 652 |
| S24 | TI ( (domicil* or home#) N3 environment* ) OR AB ( (domicil* or home#) N3 environment* ) | Expanders - Apply equivalent subjects  Search modes - Find all my search terms | 5,288 |
| S23 | TI ( (domicil* or home#) N3 dwelling ) OR AB ( (domicil* or home#) N3 dwelling ) | Expanders - Apply equivalent subjects  Search modes - Find all my search terms | 699 |
| S22 | TI ( "home-based" or homebased or "domicile-based" ) OR AB ( "home-based" or homebased or "domicile-based" ) | Expanders - Apply equivalent subjects  Search modes - Find all my search terms | 7,822 |
| S21 | TI homecare OR AB homecare | Expanders - Apply equivalent subjects  Search modes - Find all my search terms | 1,100 |
| S20 | TI ( (domicil* or home#) N3 model* ) OR AB ( (domicil* or home#) N3 model* ) | Expanders - Apply equivalent subjects  Search modes - Find all my search terms | 2,038 |
| S19 | TI ( (domicil* or home#) N3 (assist* or manag* or monitor* or network* or program* or rehab* or resourc* or servic* or support*) ) OR AB ( (domicil* or home#) N3 (assist* or manag* or monitor* or network* or program* or rehab* or resourc* or servic* or support*) ) | Expanders - Apply equivalent subjects  Search modes - Find all my search terms | 26,151 |
| S18 | TI ( (domicil* or home#) N3 (care or "health care" or healthcare) ) OR AB ( (domicil* or home#) N3 (care or "health care" or healthcare) ) | Expanders - Apply equivalent subjects  Search modes - Find all my search terms | 36,229 |
| S17 | (MH "Home Respiratory Care+") | Expanders - Apply equivalent subjects  Search modes - Find all my search terms | 1,022 |
| S16 | (MH "Home Rehabilitation+") | Expanders - Apply equivalent subjects  Search modes - Find all my search terms | 2,253 |
| S15 | (MH "Home Nursing, Professional") | Expanders - Apply equivalent subjects  Search modes - Find all my search terms | 7,444 |
| S14 | (MH "Home Health Care") | Expanders - Apply equivalent subjects  Search modes - Find all my search terms | 24,947 |
| S13 | S1 OR S2 OR S3 OR S4 OR S5 OR S6 OR S7 OR S8 OR S9 OR S10 OR S11 OR S12 | Expanders - Apply equivalent subjects  Search modes - Find all my search terms | 91,185 |
| S12 | TI ( (pulmonary or respirat*) N3 rehab* ) OR AB ( (pulmonary or respirat*) N3 rehab* ) | Expanders - Apply equivalent subjects  Search modes - Find all my search terms | 2,625 |
| S11 | (MH "Rehabilitation, Pulmonary+") | Expanders - Apply equivalent subjects  Search modes - Find all my search terms | 3,036 |
| S10 | TI respirat* N0 therap* OR AB respirat* N0 therap* | Expanders - Apply equivalent subjects  Search modes - Find all my search terms | 3,179 |
| S9 | (MH "Respiratory Therapy+") | Expanders - Apply equivalent subjects  Search modes - Find all my search terms | 52,648 |
| S8 | TI emphysema* OR AB emphysema* | Expanders - Apply equivalent subjects  Search modes - Find all my search terms | 4,654 |
| S7 | TI chronic N2 bronchitis OR AB chronic N2 bronchitis | Expanders - Apply equivalent subjects  Search modes - Find all my search terms | 1,252 |
| S6 | TI ( COPD or COAD ) OR AB ( COPD or COAD ) | Expanders - Apply equivalent subjects  Search modes - Find all my search terms | 17,955 |
| S5 | AB chronic N2 obstructi* AND AB ( ( pulmonary or airway* or (air N0 way*) or lung or lungs or airflow* or (air N0 flow*) ) | Expanders - Apply equivalent subjects  Search modes - Find all my search terms | 15,031 |
| S4 | TI chronic N2 obstructi* AND TI ( ( pulmonary or airway* or (air N0 way*) or lung or lungs or airflow* or (air N0 flow*) ) | Expanders - Apply equivalent subjects  Search modes - Find all my search terms | 8,261 |
| S3 | (MH "Emphysema") | Expanders - Apply equivalent subjects  Search modes - Find all my search terms | 2,993 |
| S2 | (MH "Pulmonary Disease, Chronic Obstructive+") | Expanders - Apply equivalent subjects  Search modes - Find all my search terms | 21,168 |
| S1 | (MH "Lung Diseases, Obstructive") | Expanders - Apply equivalent subjects  Search modes - Find all my search terms | 4,506 |
|  |  |  |  |
|  |  |  |  |
|  |  |  |  |
|  |  |  |  |
|  |  |  |  |
|  |  |  |  |
|  |  |  |  |
|  |  |  |  |
|  |  |  |  |
|  |  |  |  |
|  |  |  |  |
|  |  |  |  |
|  |  |  |  |
|  |  |  |  |
|  |  |  |  |
|  |  |  |  |
|  |  |  |  |
|  |  |  |  |
|  |  |  |  |
|  |  |  |  |
|  |  |  |  |
|  |  |  |  |
|  |  |  |  |
|  |  |  |  |
|  |  |  |  |
|  |  |  |  |
|  |  |  |  |
|  |  |  |  |
|  |  |  |  |
|  |  |  |  |
|  |  |  |  |
|  |  |  |  |
|  |  |  |  |
|  |  |  |  |
|  |  |  |  |
|  |  |  |  |
|  |  |  |  |
|  |  |  |  |
|  |  |  |  |
|  |  |  |  |
|  |  |  |  |
|  |  |  |  |
|  |  |  |  |
|  |  |  |  |
|  |  |  |  |

****************************

EconLit

| # | Query | Limiters/Expanders | Results |
| --- | --- | --- | --- |
| S31 | S8 AND S29 | Limiters - Published Date: 20200101-20211231  Expanders - Apply equivalent subjects  Search modes - Find all my search terms | 2 |
| S30 | S8 AND S29 | Expanders - Apply equivalent subjects  Search modes - Find all my search terms | 6 |
| S29 | S9 OR S10 OR S11 OR S12 OR S13 OR S14 OR S15 OR S16 OR S17 OR S18 OR S19 OR S20 OR S21 OR S22 OR S23 OR S24 OR S25 OR S26 OR S27 OR S28 | Expanders - Apply equivalent subjects  Search modes - Find all my search terms | 20,785 |
| S28 | TI ( (care or manag*) N2 themsel* ) OR AB ( (care or manag*) N2 themsel* ) | Expanders - Apply equivalent subjects  Search modes - Find all my search terms | 162 |
| S27 | TI ( (care or manag*) N2 herself ) OR AB ( (care or manag*) N2 herself ) | Expanders - Apply equivalent subjects  Search modes - Find all my search terms | 2 |
| S26 | TI ( (care or manag*) N2 himself ) OR AB ( (care or manag*) N2 himself ) | Expanders - Apply equivalent subjects  Search modes - Find all my search terms | 8 |
| S25 | TI ( (care or manag*) N2 self ) OR AB ( (care or manag*) N2 self ) | Expanders - Apply equivalent subjects  Search modes - Find all my search terms | 931 |
| S24 | TI home# | Expanders - Apply equivalent subjects  Search modes - Find all my search terms | 5,022 |
| S23 | TI HBPR OR AB HBPR | Expanders - Apply equivalent subjects  Search modes - Find all my search terms | 0 |
| S22 | TI home# N2 PR OR AB home# N2 PR | Expanders - Apply equivalent subjects  Search modes - Find all my search terms | 1 |
| S21 | TI home# N2 domicil* OR AB home# N2 domicil* | Expanders - Apply equivalent subjects  Search modes - Find all my search terms | 6 |
| S20 | TI ( (patient* N0 home#) or (patient* N0 domicil*) ) OR AB ( (patient* N0 home#) or (patient* N0 domicil*) ) | Expanders - Apply equivalent subjects  Search modes - Find all my search terms | 11 |
| S19 | TI "in home" OR AB "in home" | Expanders - Apply equivalent subjects  Search modes - Find all my search terms | 19,193 |
| S18 | TI "at home" OR AB "at home" | Expanders - Apply equivalent subjects  Search modes - Find all my search terms | 19,193 |
| S17 | TI home N0 nurs* OR AB home N0 nurs* | Expanders - Apply equivalent subjects  Search modes - Find all my search terms | 627 |
| S16 | TI ( (domicil* or home#) N3 setting# ) OR AB ( (domicil* or home#) N3 setting# ) | Expanders - Apply equivalent subjects  Search modes - Find all my search terms | 74 |
| S15 | TI ( (domicil* or home#) N3 session# ) OR AB ( (domicil* or home#) N3 session# ) | Expanders - Apply equivalent subjects  Search modes - Find all my search terms | 1 |
| S14 | TI ( (domicil* or home#) N3 dwelling ) OR AB ( (domicil* or home#) N3 dwelling ) | Expanders - Apply equivalent subjects  Search modes - Find all my search terms | 25 |
| S13 | TI ( "home-based" or homebased or "domicile-based" ) OR AB ( "home-based" or homebased or "domicile-based" ) | Expanders - Apply equivalent subjects  Search modes - Find all my search terms | 368 |
| S12 | TI homecare OR AB homecare | Expanders - Apply equivalent subjects  Search modes - Find all my search terms | 28 |
| S11 | TI ( (domicil* or home#) N3 model* ) OR AB ( (domicil* or home#) N3 model* ) | Expanders - Apply equivalent subjects  Search modes - Find all my search terms | 471 |
| S10 | TI ( (domicil* or home#) N3 (assist* or manag* or monitor* or network* or program* or rehab* or resourc* or servic* or support*) ) OR AB ( (domicil* or home#) N3 (assist* or manag* or monitor* or network* or program* or rehab* or resourc* or servic* or support*) ) | Expanders - Apply equivalent subjects  Search modes - Find all my search terms | 1,114 |
| S9 | TI ( (domicil* or home#) N3 (care or "health care" or healthcare) ) OR AB ( (domicil* or home#) N3 (care or "health care" or healthcare) ) | Expanders - Apply equivalent subjects  Search modes - Find all my search terms | 810 |
| S8 | S1 OR S2 OR S3 OR S4 OR S5 OR S6 OR S7 | Expanders - Apply equivalent subjects  Search modes - Find all my search terms | 84 |
| S7 | TI ( (pulmonary or respirat*) N3 rehab* ) OR AB ( (pulmonary or respirat*) N3 rehab* ) | Expanders - Apply equivalent subjects  Search modes - Find all my search terms | 4 |
| S6 | TI respirat* N0 therap* OR AB respirat* N0 therap* | Expanders - Apply equivalent subjects  Search modes - Find all my search terms | 1 |
| S5 | TI emphysema* OR AB emphysema* | Expanders - Apply equivalent subjects  Search modes - Find all my search terms | 4 |
| S4 | TI chronic N2 bronchitis OR AB chronic N2 bronchitis | Expanders - Apply equivalent subjects  Search modes - Find all my search terms | 15 |
| S3 | TI ( COPD or COAD ) OR AB ( COPD or COAD ) | Expanders - Apply equivalent subjects  Search modes - Find all my search terms | 44 |
| S2 | AB chronic N2 obstructi* AND AB ( ( pulmonary or airway* or (air N0 way*) or lung or lungs or airflow* or (air N0 flow*) ) | Expanders - Apply equivalent subjects  Search modes - Find all my search terms | 45 |
| S1 | TI chronic N2 obstructi* AND TI ( ( pulmonary or airway* or (air N0 way*) or lung or lungs or airflow* or (air N0 flow*) ) | Expanders - Apply equivalent subjects  Search modes - Find all my search terms | 18 |

****************************

**Web of Science**

| # 15 | [316](https://www-webofscience-com.login.ezproxy.library.ualberta.ca/wos/alldb/summary/1c5b7ad1-1b23-4ed5-8eaa-ca0c45710cfa-13cb1f66/relevance/1) | (#3) AND #13 and 2021 or 2022 (Publication Years) and English (Languages) |
| --- | --- | --- |
| # 14 | [4,614](https://www-webofscience-com.login.ezproxy.library.ualberta.ca/wos/alldb/summary/a0420883-0034-4e57-a932-a5e843b9b6d0-13cb1c70/relevance/1) | (#3) AND #13 |
| # 13 | [293,357](https://www-webofscience-com.login.ezproxy.library.ualberta.ca/wos/alldb/summary/b166a067-f004-44bf-938e-5b2a67d1dfb8-13cb1941/relevance/1) | ((((((((#4) OR #5) OR #6) OR #7) OR #8) OR #9) OR #10) OR #11) OR #12 |
| # 12 | [84,402](https://www-webofscience-com.login.ezproxy.library.ualberta.ca/wos/alldb/summary/0b848f67-e033-454e-890d-d4f9fe696d2a-13cb14bb/relevance/1) | (care or manag*) NEAR/2 self (Topic) or (care or manag*) NEAR/2 himself (Topic) or (care or manag*) NEAR/2 herself (Topic) or (care or manag*) NEAR/2 themsel* (Topic) |
| # 11 | [190](https://www-webofscience-com.login.ezproxy.library.ualberta.ca/wos/alldb/summary/86b01d1e-e5b0-4f4b-889c-699f7535621d-13cb0f6f/relevance/1) | home* NEAR/2 PR (Topic) or HBPR (Topic) |
| # 10 | [23,085](https://www-webofscience-com.login.ezproxy.library.ualberta.ca/wos/alldb/summary/23209e14-7dee-44b5-9103-964060443155-13cb0dd2/relevance/1) | patient* NEAR/2 (home or homes or domicil*) (Topic) or (home or homes) NEAR/2 domicil* (Topic) |
| # 9 | [95,829](https://www-webofscience-com.login.ezproxy.library.ualberta.ca/wos/alldb/summary/f0fbbbd8-fdf5-4dd6-8a1a-72c63aae21b4-13cb08d7/relevance/1) | "at home" (Topic) or "in home" (Topic) |
| # 8 | [5,531](https://www-webofscience-com.login.ezproxy.library.ualberta.ca/wos/alldb/summary/828a8665-69d3-4b1e-8d42-39b7236da82d-13cb057b/relevance/1) | "home nursing" (Topic) or "home nurse" (Topic) or "home nursed" (Topic) or "home nurses" (Topic) |
| # 7 | [30,319](https://www-webofscience-com.login.ezproxy.library.ualberta.ca/wos/alldb/summary/483ca486-d0ae-4459-a29c-5c203f9a1f73-13cb0022/relevance/1) | (domicil* or home or homes) NEAR/3 dwelling (Topic) or (domicil* or home or homes) NEAR/3 environment* (Topic) or (domicil* or home or homes) NEAR/3 (session or sessions) (Topic) or (domicil* or home or homes) NEAR/3 (setting or settings) (Topic) |
| # 6 | [17,073](https://www-webofscience-com.login.ezproxy.library.ualberta.ca/wos/alldb/summary/62d78bbd-fa22-4198-95cd-6ce1b54cca10-13caf900/relevance/1) | "home-based" or homebased or "domicile-based" (Topic) |
| # 5 | [1,782](https://www-webofscience-com.login.ezproxy.library.ualberta.ca/wos/alldb/summary/1da892bb-430f-40b4-92e2-75abd15d913a-13caf7df/relevance/1) | homecare (Topic) |
| # 4 | [102,917](https://www-webofscience-com.login.ezproxy.library.ualberta.ca/wos/alldb/summary/c4a455b4-9cc5-4ad9-9c6d-9d549f69c826-13caf321/relevance/1) | (domicil* or home or homes) NEAR/3 (care or "health care" or healthcare) (Topic) or (domicil* or home or homes) NEAR/3 (assist* or manag* or monitor* or network* or program* or rehab* or resourc* or servic* or support*) (Topic) or (domicil* or home or homes) NEAR/3 model* (Topic) |
| # 3 | [155,385](https://www-webofscience-com.login.ezproxy.library.ualberta.ca/wos/alldb/summary/1636da5a-76fb-4639-9425-df731f15c86e-13caee1e/relevance/1) | #2 OR #1  Indexes=SCI-EXPANDED, SSCI, A&HCI, CPCI-S, CPCI-SSH, BKCI-S, BKCI-SSH, ESCI, CCR-EXPANDED, IC Timespan=All years |
| # 2 | [127,720](https://www-webofscience-com.login.ezproxy.library.ualberta.ca/wos/alldb/summary/4119a4f4-593e-4c58-a243-0b5542a809d4-13caec64/relevance/1) | COPD or COAD (Topic) or chronic NEAR/2 bronchitis (Topic) or emphysema* (Topic) or respirat* NEAR/0 therap* (Topic) or (pulmonary or respirat*) NEAR/3 rehab* (Topic) |
| # 1 | [85,031](https://www-webofscience-com.login.ezproxy.library.ualberta.ca/wos/alldb/summary/38fa0534-c5b1-4f83-97bf-9257db297275-13cae6a7/relevance/1) | TS=(chronic NEAR/2 obstructi*) AND TS=(pulmonary or airway* or (air NEAR/0 way*) or lung or lungs or airflow* or (air NEAR/0 flow*) ) |

| Supplementary Appendix 2. Summary of included studies of home-based pulmonary rehabilitation | | | | | | | |
| --- | --- | --- | --- | --- | --- | --- | --- |
| **Study**  **(country)** | **Study period**  **(Design)** | **Study objective** | **Eligibility criteria** | **Number of centres** | **Number of participants** | **Follow-up** | **Outcomes** |
| ***HBPR vs ‘usual care’*** | | | | | | | |
| Lahham 2019  (Australia) | Apr 2015- Nov 2017  (RCT) | To determine the effectiveness of HBPR in patients with mild COPD. | *Inclusion criteria:*   - Age >40 years old - Mild COPD (FEV_1_/FVC<70%, FEV_1_ ≥80% predicted) - Smoking history of ≥10 pack years   *Exclusion criteria:*   - Acute exacerbation in last month - Hospitalization in month previous to recruitment - Asthma - Restrictive Lung Disease - Primary Diagnosis of other respiratory related disease - Comorbidities preventing exercise participation | Multiple centres | HBPR: 29  Usual care: 29 | 6 months | - Adherence - Dyspnea - Functional exercise capacity - Health-related quality of life |
| Coultas 2018  USA | Apr 2010- Apr 2014  (RCT) | To examine the effectiveness of a behavioral lifestyle physical activity intervention combined with chronic obstructive pulmonary disease self-management education to prevent high-cost health care utilization. | *Inclusion criteria:*   - Age ≥45 years old - Physician-diagnosed and spirometry-confirmed COPD (FEV1/FVC<70%, FEV1 70% predicted) - mMRC score≥2   *Exclusion criteria:*   - Attended PR in the previous year - Nursing home resident - Uncontrolled hypertension, angina, heart failure - Unstable EKG findings - Dementia, uncontrolled psychiatric illness - Life expectancy <12months - Resting oxygen saturation <90% and inability to obtain supplemental oxygen - 6 minute walk <110m - Other safety concerns with participating in physical activity | Single centre | HBPR: 149  Usual care: 156 | 18 months | - Functional exercise capacity - Hospital admission - Health-related quality of life - Safety |
| Li 2018  (China) | Jun 2014- Apr 2016  (RCT) | To compare a home-visit strategy to usual care  in maintaining PR benefits. | *Inclusion criteria:*   - Diagnosis of COPD based on GOLD guidelines - Clinically stable in the last month - Able to complete the PR program and questionnaire survey successfully and independently   *Exclusion criteria:*   - Asthma - Obstructive sleep apnea syndrome - Cancer - Diagnosis of Alzheimer’s disease or depression and anxious disorder - Incapable of exercising even during the stable stage - Severe dysfunction of the heart, liver, or kidney - Suffering emotional trauma in the previous 6 months such as relative death and divorce | Single centre | HBPR: 82  Usual care: 69 | 12 months | - Adherence - Exacerbation - Functional exercise capacity - Mental health - Health-related quality of life - Safety |
| Khoshkesht 2015  (Iran) | Dec 2010- Feb 2011  (RCT) | To investigate the effect of pulmonary rehabilitation on the self-efficacy of patients with chronic obstructive pulmonary disease. | *Inclusion criteria:*   - Age<65 years old - Mild or moderate COPD (FEV_1_/FVC<70%, FEV_1_ ≥50% predicted) - Able to read, write and speak Persian   *Exclusion criteria:*   - Attended PR in the previous year - Cardiac, musculoskeletal and mental diseases that could interfere with exercise - Recent exacerbation of the disease - Participated in any sort of formal exercise training - Advised by their treatment teams to restrict their mobility - Unable to follow nutritional recommendations and exercise programmes | Single centre | HBPR: 35  Usual care: 35 | 7 weeks | - Adherence - Self-efficacy |
| Pradella 2015  (Brazil) | NR  (RCT) | To assess the functional exercise capacity and quality of life of HBPR. | *Inclusion criteria:*   - Age≥40 years old - Diagnosis of COPD based on the criteria of the Brazilian Thoracic Society and GOLD (FEV1 < 80% of predicted)   *Exclusion criteria:*   - Age≥75 years old - Attended PR in the previous year - Regular practice of any type of physical activity (e.g. fast walking, swimming) for at least 40 min, 3 times/week, in the previous 12 months - Presence of other disease that could lead to exercise intolerance, such as neurological, heart, or orthopedic conditions - Cognitive impairment - Exacerbation in month previous to recruitment | Single centre | HBPR: 32  Usual care: 18 | 8 weeks | - Adherence - Functional exercise capacity - Lung function - Health-related quality of life - Safety |
| De Sousa Pinto 2014  (Spain) | Oct 2009- Jun 2011  (RCT) | To assess the effectiveness of HBPR on quality of life and functional capacity outcomes in patients with COPD. | *Inclusion criteria:*   - Age ≤ 80 years - Severe or very severe COPD with or without oxygen therapy - Former smokers   *Exclusion criteria:*   - Previously attended PR - Long exacerbation periods - Cognitive problems, musculoskeletal disorders, cardiac disease, or other disabling diseases that could restrict exercising, and communication problems | Single centre | HBPR: 29  Usual care: 21 | 12 weeks | - Adherence - Dyspnea - Functional exercise capacity - Health-related quality of life |
| Liu 2013  (China) | Dec 2009- Oct 2011  (RCT) | To evaluate the effectiveness of an online breathing program on pulmonary function, exercise capacity, and health-related quality of life in patients with COPD. | *Inclusion criteria:*   - Diagnosis of COPD based on the Chinese Society of Respiratory Disease guidelines - Stable clinical condition - Access to a computer with Internet access in the home   *Exclusion criteria:*   - History of bronchial asthma - Bronchiectasis - Oral glucocorticoid treat­ment within the previous three months - Cancer - Cardiac failure - Distal arteriopathy - Severe endocrine, hepatic, or renal disease | Single centre | HBPR: 30  Usual care: 30 | 4 months | - Adherence - Exacerbation - Functional exercise capacity - Health-related quality of life - Safety |
| Mendes de Oliveira 2010  (Brazil) | Jan 2007- May 2009  (RCT) | To compare the effectiveness of HBPR and OPR. | *Inclusion criteria:*   - Diagnosis of COPD based on GOLD guidelines - Clinically stable in the last 2 months (no reports of worsening of dyspnea, increased phlegm production or phlegm purulence)   *Exclusion criteria:*   - Hospitalization - Presence of neuromuscular disease, associated respiratory disease, orthopedic or neurological disease that affected gait - Recent impairment due to comorbidities, such as myocardial infarction, heart failure, stroke or neoplasm - Prior pneumonectomy or other thoracic surgery. | Single centre | HBPR: 42  Usual care: 29 | 12 weeks | - Adherence - Functional exercise capacity |
| Moore 2009  (UK) | NR  (RCT) | To determine a home exercise program could improve walking ability, breathlessness and quality of life. | *Inclusion criteria:*   - Diagnosis of COPD (FEV1/FVC<70%, FEV1 <60% predicted) - Access to a Video Home System or Digital Video Disc player   *Exclusion criteria:*   - Previously attended PR - Comorbid conditions that would preclude safe exercise training at home - Significant cardiac disease and cognitive impairments - High blood pressure (> 180/100) - High heart rate (> 100) - Low oxygen saturations (< 88%) | Single centre | HBPR: 14  Usual care: 13 | Mean±SD  HBPR: 8±3 weeks  no PR: 7±1 weeks | - Adherence - Exacerbation - Functional exercise capacity - Health-related quality of life |
| Lalmolda 2017  (Spain) | Jan 2011- NR  Cohort study | To assess the effectiveness of home-based exercise after a severe exacerbation on health care utilization. | *Inclusion criteria:*   - Age >40 years old - Diagnosis of COPD based on the ATS/ ERS guidelines - Admitted to hospital for an exacerbation in the last three years   *Exclusion criteria:*   - One or more comorbidities limiting cognitive or exercise capabilities - A cluster of 3 admissions in the last year - Admission >30 days - Asthma, cystic fibrosis, bronchiectasis - Cancer - Long-term treatment with oral corticosteroids or immunosuppressors | Multiple centres | HBPR: 21  Usual care: 29 | 12 months | - Adherence - Exacerbation - Functional exercise capacity - Hospital admission - Length of hospitalization - Safety |
| ***HBPR vs OPR*** | | | | | | | |
| Horton 2017  (UK) | Nov 2007- Jul 2012  (RCT) | To compare HBPR and OPR. | *Inclusion criteria:*   - Diagnosis of COPD - MRC≥2 - Proficiency in English   *Exclusion criteria:*   - Attended PR in the previous year - Lower limb disability and other unstable comorbid conditions | Single centre | HBPR: 145  OPR: 142 | 6 months | - Adherence - Functional exercise capacity - Hospital admission - Mental health - Health-related quality of life - Safety - Self-efficacy |
| Holland 2017  (Australia) | Oct 2011- May 2015  (RCT) | To compare costs and clinical benefits between HBPR and OPR. | *Inclusion criteria:*   - Age >40 years old - Diagnosis of COPD (FEV1/FVC<70%) - Smoking history of ≥10 pack years   *Exclusion criteria:*   - Attended PR in the last two years - Acute exacerbation in the previous month - Asthma - Comorbidities preventing exercise participation | Multiple centres | HBPR: 80  OPR: 86 | 12 months | - Adherence - Dyspnea - Functional exercise capacity - Hospital admission - Length of hospitalization - Health-related quality of life - Safety - Self-efficacy |
| Mendes de Oliveira 2010  (Brazil) | Jan 2007- May 2009  (RCT) | To compare the effectiveness of HBPR and OPR. | *Inclusion criteria:*   - Diagnosis of COPD based on GOLD guidelines - Clinically stable in the last 2 months (no reports of worsening of dyspnea, increased phlegm production or phlegm purulence)   *Exclusion criteria:*   - Hospitalization - Presence of neuromuscular disease, associated respiratory disease, orthopedic or neurological disease that affected gait - Recent impairment due to comorbidities, such as myocardial infarction, heart failure, stroke or neoplasm - Prior pneumonectomy or other thoracic surgery. | Single centre | HBPR: 42  OPR: 46 | 12 weeks | - Adherence - Functional exercise capacity |
| Nolan 2019  (UK) | 2012- 2015  (Cohort study) | To assess the effectiveness of home-based exercise compared with OPR. | *Inclusion criteria:*   - Physician diagnosis of COPD referred to PR   *Exclusion criteria:*   - Comorbidities that would make exercise unsafe (e.g. unstable angina, severe aortic stenosis, abdominal aortic aneurysm >5.5cm, uncontrolled cardiac arrhythmias) | Single centre | HBPR: 154  OPR: 154 | 8 weeks | - Adherence - Functional exercise capacity - Health-related quality of life |
| Chaplin 2017  (UK) | May 2013- Jul 2015  (RCT) | To examine if a web-based rehabilitation programme could be an alternative to conventional rehabilitation programme. | *Inclusion criteria:*   - Diagnosis of COPD (FEV1/FVC<70%, FEV_1_< 80% predicted) - MRC≥2 - Access to the internet for more than 3 months - Able to navigate around websites and regular use of email - Able to read and write in English   *Exclusion criteria:*   - Attended PR in the previous year - Comorbidities that would make exercise unsafe | Multiple centres | HBPR: 51  OPR: 52 | Mean±SD  HBPR: 11±4 weeks  OPR: NR | - Adherence - Functional exercise capacity - Mental health - Health-related quality of life - Self-efficacy |
| Notes: No pulmonary rehabilitation (Usual care): patients were managed by their GP, specialist or both according to local practices.  ATS: American Thoracic Society; COPD: chronic obstructive pulmonary disease; EKG: electrocardiogram; ERS: European Respiratory Society; FEV_1_: forced expiratory volume in one second; FVC: forced vital capacity; HBPR: home-based pulmonary rehabilitation; mMRC: Modified Medical Research Council; MRC: Medical Research Council; NR: not reported; OPR: outpatient pulmonary rehabilitation; PR: pulmonary rehabilitation; RCT: randomized controlled trial; SD: standard deviation | | | | | | | |

| Supplementary Appendix 3. Characteristics of patient populations across included studies | | | | | | | | | | | | | |
| --- | --- | --- | --- | --- | --- | --- | --- | --- | --- | --- | --- | --- | --- |
| **Study** | **Sample size** | **Age in years**  **Mean±SD**  **Median (IQR)** | **Gender n(% Male)** | **BMI in kg/m^2^**  **Mean±SD** | **COPD GOLD stages**  **n (%)** | **FEV_1_ in % predicted**  **Mean±SD**  **Median (IQR)** | **FEV_1_ in l**  **Mean±SD**  **Median (IQR)** | **FVC (l)**  **Mean±SD**  **Median (IQR)** | **FEV_1_/FVC ratio in %**  **Mean±SD**  **Median (IQR)** | **Comorbidities**  **Mean±SD**  **Median (IQR)** | **Smoking status**  **N (%)** | **LTOT**  **N(%)** |  |
| ***HBPR vs ‘usual care’*** | | | | | | | | | | | | | |
| Lahham 2019  Australia | HBPR: 29  Usual care: 29 | HBPR: 68.0± 9.0  Usual care: 67.0±10.0  p=NR | HBPR: 17 (59)  Usual care: 17 (59)  p=NR | HBPR: 28.0±4.5  Usual care: 28.0±4.3  p=NR | HBPR:  Mild: 29 (100)  Moderate: 0 (0)  Severe: 0 (0)  Very severe: 0 (0)  Usual care:  Mild: 29 (100)  Moderate: 0 (0)  Severe: 0 (0)  Very severe: 0 (0)  p=NR | HBPR: 90± 8  Usual care: 92±7  p=NR | HBPR: 2.4±0.6  Usual care: 2.7±0.6  p=NR | HBPR: 3.8±0.9  Usual care: 4.3±1.1  p=NR | HBPR: 57±20  Usual care: 61±13  p=NR | NR | HBPR:  Current: 6(21)  Former: 23 (79)  Never: 0 (0)  Usual care:  Current: 6(21)  Former: 23 (79)  Never: 0 (0)  p=NR | HBPR: 0 (0)  Usual care: 0 (0)  p=NA |  |
| Coultas 2018  USA | HBPR: 149  Usual care: 156 | HBPR: 70.8±9.5  Usual care: 69.8±9.5  p>0.05 | HBPR: 74 (50)  Usual care: 77 (49)  p>0.05 | NR | HBPR:  Mild: 0 (0)  Moderate: 59 (40)  Severe: 71 (48)  Very severe: 19 (13)  Usual care:  Mild: 0 (0)  Moderate: 75 (48)  Severe: 59 (38)  Very severe: 22 (14)  p>0.05 | HBPR: 45.5±12.6  Usual care: 47.3±13.5  p>0.05 | NR | NR | NR | HBPR: 3.1±2.2  5 (4-6)  Usual care: 2.9±1.7  5 (4-6)  p>0.05 | HBPR:  Current: 30 (20)  Former: 107 (72)  Never: 12 (8)  Usual care:  Current: 46 (29)  Former: 99 (63)  Never: 11 (7)  p>0.05 | HBPR: 6 (4)  Usual care: 15 (10)  p>0.05 |  |
| Li 2018  China | HBPR: 82  Usual care: 69 | HBPR: 65.1±8.7  Usual care: 66.0±9.3  p=0.19 | HBPR: 71 (87)  Usual care: 55 (80)  p=0.28 | HBPR: 24.0±2.0  Usual care: 24.4±2.0  p=NR | NR | HBPR: 49.2±12.2  Usual care: 49.1±11.1  p=NR | HBPR: 1.1±0.4  Usual care: 1.1±0.4  p=NR | HBPR: 2.3±0.5  Usual care: 2.3±0.5  p=NR | HBPR: 49.5±11.4  Usual care: 48.8±10.3  p=NR | NR | NR | NR |  |
| Khoshkesht 2015  Iran | HBPR: 35  Usual care: 35 | NR | HBPR(n=34): 24(71)  Usual care(n=32): 23(72)  p=0.91 | NR | HBPR(n=34):  Mild: 15(44)  Moderate: 19(56)  Severe: 0 (0)  Very severe: 0 (0)  Usual care(n=32):  Mild: 14(44)  Moderate: 18(56)  Severe: 0 (0)  Very severe: 0 (0)  p=0.98 | NR | NR | NR | NR | NR | HBPR (n=34):  Current/former: 24(71)  Never: 10(29)  Usual care (n=32):  Current/ former: 23 (72)  Never: 9 (28)  p=0.90 | NR |  |
| Pradella 2015  Brazil | HBPR: 32  Usual care: 18 | HBPR(n=29):  62.4±10.7  Usual care(n=15): 65.3±8.0  p>0.05 | HBPR(n=29): 23 (79)  Usual care(n=15): 13 (87)  p=NR | HBPR(n=29): 25.2±5.0  Usual care(n=15): 26.7±5.3  p=NR | HBPR(n=29):  Mild: 1 (3)  Moderate: 6 (21)  Severe: 14 (48)  Very severe: 8 (28)  Usual care(n=15):  Mild: 1 (7)  Moderate: 8 (53)  Severe: 3 (20)  Very severe: 3 (20)  p=NR | HBPR(n=29): 43.9±16.2  Usual care(n=15): 54.0±22.2  p=NR | HBPR(n=29): 1.2± 0.4  Usual care(n=15): 1.5±0.6  p>0.05 | HBPR (n=29): 2.8+0.8  Usual care (n=15): 3.1+0.7  p>0.05 | HBPR(n=29):45±13  Usual care (n=15): 48±14  p=NR | NR | NR | NR |  |
| De Sousa Pinto 2014  Spain | HBPR: 29  Usual care: 21 | HBPR(n=23):  68.9±9.2  Usual care(n=18): 71.9±7.6  p>0.05 | HBPR(n=23): 22 (96)  Usual care(n=18): 17 (94)  p>0.05 | HBPR(n=23): 26.0±3.3  Usual care(n=18): 26.4±5.0  p>0.05 | HBPR(n=23):  Mild: 0 (0)  Moderate: 0 (0)  Severe: 9 (39)  Very severe: 14 (61)  Usual care(n=18):  Mild: 0 (0)  Moderate: 0 (0)  Severe: 10 (56)  Very severe: 8 (44)  p>=0.05 | HBPR(n=23): 33.5±7.3  Usual care(n=18): 34.5±9.5  p>0.05 | HBPR(n=23): 0.9±0.2  Usual care(n=18): 0.9±0.2  p>0.05 | HBPR(n=23): 2.5±0.8  Usual care(n=18): 2.3±0.5  p>0.05 | HBPR(n=23): 74.4±18.9  Usual care(n=18): 67.9±16.4  p>0.05 | NR | NR | NR |  |
| Liu 2013  China | HBPR: 30  Usual care: 30 | HBPR(n=29): 69.4±3.3  Usual care(n=28): 68.8±1.4  p=0.87 | HBPR(n=29): 21 (72)  Usual care(n=28): 23 (82)  p=0.53 | HBPR(n=29): 19.2 ±0.4  Usual care(n=28): 18.8±0.2  p=0.50 | HBPR:  Mild: 0 (0)  Moderate: 19 (63)  Severe: 11 (37)  Very severe: 0 (0)  Usual care:  Mild: 0 (0)  Moderate: 17 (57)  Severe:13 (43)  Very severe: 0 (0)  p=NR | HBPR(n=29): 49.2± 0.5  Usual care(n=28): 49.8± 0.7  p=0.56 | HBPR(n=29): 0.9±0.1  Usual care(n=28): 1.0±0.0  p=0.27 | HBPR(n=29): 1.7±0.0  Usual care(n=28): 1.8±0.0  p=0.18 | HBPR(n=29): 52.6±0.5  Usual care (n=28): 53.4± 0.4  p=0.58 | NR | HBPR (n=29):  Current: 10 (34)  Former: 14 (14)  Never: 5 (17)  Usual care (n=28):  Current: 8 (29)  Former: 12 (43)  Never: 8 (29)  p=0.43 | NR |  |
| Mendes de Oliveira 2010  Brazil | HBPR: 42  Usual care: 29 | HBPR(n=33): 66.4±9.5  Usual care(n=29): 70.8±8.7  p=NR | HBPR(n=33): 27 (82)  Usual care(n=29): 19 (66)  p=NR | HBPR(n=33): 25.0±6.2  Usual care(n=29): 24.6±6.3  p=NR | HBPR(n=33):  Mild: 4 (12)  Moderate: 8 (24)  Severe: 11 (33)  Very severe: 10 (30)  Usual care(n=29):  Mild: 2 (7)  Moderate: 6 (21)  Severe: 13 (45)  Very severe: 8 (28)  p=NR | HBPR(n=33): 47.5±23.3  Usual care(n=29): 41.4±18.4  p=NR | HBPR(n=33): 1.3±0.8  Usual care(n=29): 1.0 ± 0.5  p=NR | HBPR(n=33): 2.2±0.7  Usual care(n=29): 2.2± 0.5  p=NR | HBPR(n=33): 69.1±21.8  Usual care(n=29): 59.2±20.6  p=NR | NR | Ex and current  HBPR (n=33):  Current/former: 32 (97)  Never: 1 (3)  Usual care (n=29):  Current/former: 28 (97)  Never: 1 (3)  p=NR | NR |  |
| Moore 2009  UK | HBPR: 14  Usual care: 13 | HBPR (n=10): 70 (NR)  Usual care (n=10): 70.5 (57.5- 78.5)  p>0.05 | HBPR (n=10): 6 (60)  Usual care (n=10): 4 (40)  p=0.66 | NR | NR | HBPR (n=10):  40.0 (36.5 49.0)  Usual care (n=10): 41.5 (30.0-55.0)  p>0.05 | HBPR (n=10): 0.9 (0.7-1.2)  Usual care (n=10): 1.0 (0.8-1.3)  p>0.05 | NR | NR | NR | NR | NR |  |
| Lalmolda 2017  Spain | HBPR: 21  Usual care: 29 | HBPR (n=19): 74.3±7.6  Usual care: 71.4±6.8  p=0.18 | HBPR (n=19): 18 (95)  Usual care: 27 (93)  p=1.00 | NR | NR | HBPR (n=19): 46.7±10.6  Usual care:  45.4±14.7  p=0.74 | NR | NR | NR | HBPR (n=19): 2 (1–3)  Usual care: 2 (1–3)  p=0.82 | HBPR (n=19):  Current: 4 (21)  Former: NR  Never: NR  Usual care:  Current: 4 (14)  Former: NR  Never: NR  p=0.69 | NR |  |
| ***HBPR vs OPR*** | | | | | | | | | | | | | |
| Horton 2017  UK | HBPR: 145  OPR: 142 | HBPR: 68±9  OPR: 67±8  p=NR | HBPR: 93 (64)  OPR: 94 (66)  p=NR | HBPR: 27±6  OPR: 28±6  p=NR | NR | HBPR: 47.9±18.7  OPR: 48.8±17.2  p=NR | HBPR: 1.2±0.5  OPR: 1.3±0.6  p=NR | HBPR: 2.7±0.8  OPR: 2.7±0.9  p=NR | NR | NR | HBPR:  Current: 45 (31)  Former: 92 (63)  Never: 8 (6)  OPR:  Current: 28 (20)  Former: 109 (77)  Never: 5 (4)  p=NR | HBPR: 9 (6)  OPR: 15 (11)  p=NR |  |
| Holland 2017  Australia | HBPR: 80  OPR: 86 | HBPR: 69±13  OPR: 69±10  p=NR | HBPR: 48 (60)  OPR: 51 (59) | HBPR: 29±7  OPR: 28±6  p=NR | NR | HBPR: 52±19  OPR: 49±19 | HBPR: 1.3±0.5  OPR: 1.2±0.6  p=NR | HBPR: 2.7±0.7  OPR: 2.7±0.9  p=NR | HBPR: 47±16  OPR: 45±14  NR | HBPR: 4(2-6)  OPR: 5(3-6) | HBPR:  Current: 10 (13)  Former: 70 (87)  Never: 0 (0)  OPR:  Current: 18 (21)  Former: 68 (79)  Never: 0 (0)  p=NR | HBPR: 2(3)  OPR: 6(7)  p=NR |  |
| Mendes de Oliveira 2010  Brazil | HBPR: 42  OPR: 46 | HBPR (n=33): 66.4±9.5  OPR (n=23): 71.3±6.7  p=NR | HBPR (n=33):  27 (82)  OPR (n=23): 19 (83)  p=NR | HBPR (n=33): 25.0±6.2  OPR (n=23): 23.5±4.2  p=NR | HBPR (n=33):  Mild: 4 (12)  Moderate: 8 (24)  Severe: 11 (33)  Very severe: 10 (30)  OPR (n=23):  Mild: 2 (9)  Moderate: 10 (43)  Severe: 5 (22)  Very severe: 6 (26)  p=NR | HBPR (n=33): 47.5±23.3  OPR (n=23): 51.5±23.9  p=NR | HBPR (n=33): 1.3±0.8  OPR (n=23): 1.3 ± 0.7  p=NR | HBPR(n=33): 2.2± 0.7  OPR(n=23): 2.5±0.8  p=NR | HBPR(n=33): 69.1±21.8  OPR(n=23): 65.1±24.5  p=NR | NR | HBPR (n=33):  Current/former: 32 (97)  Never: 1 (3)  OPR(n=23):  Current/ former: 20 (87)  Never: 3 (13)  p=NR | NR |  |
| Nolan 2019  UK | HBPR: 154  OPR: 154 | HBPR: 71±10  OPR: 71±9  p=0.76 | HBPR: 75 (49)  OPR: 72 (47)  p=0.82 | HBPR: 28.3±7.8  OPR: 27.9±7.2  p=0.97 | NR | HBPR: 45.7±19.7  OPR: 45.3±20.3  p=0.87 | HBPR: 1.1±0.6  OPR: 1.0±0.5  p=0.54 | NR | HBPR: 47±14  OPR: 46±13  p=0.39 | NR | HBPR:  Current: 32 (21)  Former: 105 (68)  Never: 17 (11)  OPR:  Current: 29 (19)  Former: 108 (70)  Never: 17 (11)  p=0.85 | HBPR: 17 (11%)  OPR: 12 (8%)  p=0.33 |  |
| Chaplin 2017  UK | HBPR: 51  OPR: 52 | HBPR: 66.4±10.1  OPR: 66.1±8.1  p= NR | HBPR: 38 (75)  OPR:  33 (63)  p=NR | HBPR: 27.9±6.4  OPR: 29.3±6.3  p=NR | NR | HBPR: 58.7±29.1  OPR:  55.0±20.5  p=NR | HBPR: NR  OPR: NR | NR | NR | NR | NR | NR |  |
| *Reported as n(%)  Notes: No pulmonary rehabilitation (Usual care): patients were managed by their GP, specialist or both according to local practices. COPD: chronic obstructive pulmonary disease; FEV_1_: forced expiratory volume in one second; FVC: forced vital capacity; IQR: interquartile range; LTOT: Long-term oxygen therapy; NR: not reported; OPR: outpatient pulmonary rehabilitation; PR: pulmonary | | | | | | | | | | | | | |

| Supplementary Appendix 4. Summary of HBPR comparator interventions in included studies | | | | |
| --- | --- | --- | --- | --- |
| **Study** | **Duration of HBPR Program** | **Comparator** | **HBPR Intervention** | **HBPR Program Equipment** |
| ***HBPR vs usual care*** | | | | |
| Lahham 2019  Australia | 8 weeks | - Usual Care - Patients provided an information booklet on living with COPD - Initial phone call with physiotherapist - Advised to stay active and take medication as prescribed - Weekly phone calls to control for attention - No feedback on exercise or activity progression provided | - Patients provided an information booklet on living with COPD - Initial home visit with physiotherapist - Patients provided with a home diary for monitoring - Weekly phone calls with physiotherapist - Patients provided with an exercise prescription - Unsupervised home exercise 5 times per week for 30 minutes per day - Unsupervised home exercise training included endurance, lower limb, upper limb exercises - Motivational interviewing during weekly phone calls used by physiotherapist to encourage behavior change - Patient selected from menu of educational topics to discuss during weekly phone call with physiotherapist - Weekly phone calls included disease specific self-management training | - Pedometer provided to patients to track walking distance - No special equipment required - Exercise training used daily activities and equipment available in the home |
| Coultas 2018  USA | 18 months | - Usual care (10 months) - Self-management needs assessment - Patients provided with a COPD self-management manual - Six-week self-management education program delivered by a health coach using a workbook and weekly telephone calls - Twenty weeks of Usual Care | - Self-management needs assessment - Patients provided with a COPD self-management manual - Six-week self-management education program delivered by a health coach using a workbook and weekly telephone calls - Twenty-week physical activity and behavior change program delivered by a health coach using a self-study workbook and weekly telephone calls - Ten-month maintenance program | - Computer assisted telephone system (CAT) |
| Li 2018  China | 12 months | - Usual care (12 months) - Eight-week Outpatient Pulmonary Rehabilitation Program | - Eight-week Outpatient Pulmonary Rehabilitation Program - Twelve-month Home Based Pulmonary Rehabilitation Program - Bi-weekly home visits for two months - Monthly home visit and weekly telephone calls for four months - Weekly telephone calls for six months - Patients provided with an exercise prescription - Unsupervised home exercise once per week (Supervised bi-weekly for first two months) - Unsupervised home exercise training included endurance and upper limb exercises - Unsupervised respiratory training three times per week - Health education provided during bi-weekly home visits for first two months | NR |
| Khoshkesht 2015  Iran | 7 weeks | - Usual care | - Three in person educational sessions - Unsupervised muscle stretching exercises three times per week - Unsupervised breathing exercises three to four times per day - Weekly telephone calls with nurses - Patients kept daily records on program checklists | - None required |
| Pradella 2015  Brazil | 8 weeks | - Usual care - Weekly telephone call with nurse to assess clinical status | - One week of in-person training at the rehabilitation centre - Patients provided with an educational booklet - Patients provided with a log for monitoring activities - Unsupervised exercise three times per week - Unsupervised relaxation exercise three times per week - Weekly telephone call with nurse to assess clinical status and encourage program adherence | - Platform for step-up exercises provided to patients without stairs - One kilogram oil can |
| De Sousa Pinto 2014  Spain | 12 weeks | - Usual care - In-person session with nurse to receive instruction on inhaler devices and nebulizer therapy | - In-person session with nurse to receive instruction on inhaler devices and nebulizer therapy - Twelve-week home based pulmonary rehabilitation program - Patients provided with guideline on COPD and COPD treatment - Education provided by physiotherapist in first two in-home visits - Patients provided with exercise prescription - Supervised exercise twice per week for two weeks followed by twice per month - Unsupervised exercise weekly (frequency not specified) - Weekly telephone calls | - Weights for upper and lower limb exercises |
| Liu 2013  China | 16 weeks | - Usual care - Exercise capacity assessed using six-minute walk test prior to hospital discharge - Pulmonary function assessed prior to hospital discharge - At time of hospital discharge, in-person instruction from respiratory therapy on importance of exercise and advised to perform exercises twice daily for sixteen weeks - Patients provided with handouts with pictures of breathing exercises | - Exercise capacity assessed using six-minute walk test prior to hospital discharge - Pulmonary function assessed prior to hospital discharge - Sixteen-week online home based pulmonary rehabilitation program - Fifteen-twenty minutes per session with two sessions per day - System monitored program participation - Nurses contacted patients by telephone if they were not regularly logging into the system - Patients could communicate with other patients and nurses through online platform | - Online home based pulmonary rehabilitation program |
| Mendes de Oliveira 2010  Brazil | 12 weeks | - Usual care - In-person group education session - Patients provided with written chart summarizing content covered during education session - Comprehensive assessment conducted at the clinic - Monthly telephone calls to assess health status | - In-person group education session - Patients provided with written chart summarizing content covered during education session - Comprehensive assessment conducted at the clinic - Exercise prescription provided - In-person exercise program training at the clinic - Home exercise program three times per week for twelve weeks - Patients recorded exercise activities in a log after each exercise session - Weekly telephone calls from health care provider | - Hand and ankle weights - Heart rate monitor |
| Moore 2009  UK | 6 weeks | - Usual care - In-person consultation with a physiotherapist - Patients provided with an educational booklet | - In-person consultation with a physiotherapist - Patients provided with an educational booklet - Patients watched a video on the benefits of exercise with a physiotherapist in-person at the clinic - Patients were provided with an exercise video to take home - Home exercise program four times a week for six weeks - Patients recorded daily exercise in a diary | - None required |
| Lalmolda 2017  Spain | 8 weeks | - Usual care | - Education on prescribed oral and inhaled medications delivered to patients in person prior to hospital discharge - Current smokers referred to smoking cessation program - Supervised home-based exercise program delivered by physiotherapist for one hour twice a week for eight weeks - Functional exercise and respiratory capacity assessed by physiotherapist - Individualized daily exercise prescription provided to patient by physiotherapist after the first week - Compliance with daily home exercises reviewed with physiotherapist using a checklist at each supervised exercise session - Ongoing compliance with unsupervised exercise monitored through monthly follow-up telephone calls with nurses for ten months | - None required |
| ***HBPR vs OPR*** | | | | |
| Horton 2017  UK | 7 weeks | - Outpatient pulmonary rehabilitation program two days a week for seven weeks - Sessions were two hours each and included exercise and education | - Introductory educational session in-person at the hospital - Exercise capacity assessed in-person at the hospital - Patients provided with an exercise prescription - Patients provided with a standardized manual to support a home exercise program - Unsupervised exercise program at home with daily endurance training and strength training three times per week for seven weeks - Patients provided with exercise log to track daily physical activity - Telephone calls during week two and week four to monitor patient progress and encourage patients | - None required |
| Holland 2017  Australia | 8 weeks | - Outpatient pulmonary rehabilitation program two days a week for eight weeks - Group sessions were supervised and included exercise and education - Patients were provided with an exercise prescription - Supervised exercise included thirty minutes of aerobic exercise and lower and upper limb strength training - Self-management education was both structured and unstructured - Patients were encouraged to exercise at home an additional three days per week - Patients recorded home exercise in a home diary and reviewed the diary with the multi-disciplinary team weekly | - Patients provided an information booklet on living with COPD - Initial home visit with physiotherapist - Patients provided with a home diary for monitoring - Weekly phone calls with physiotherapist - Patients provided with an exercise prescription - Unsupervised home exercise 5 times per week for 30 minutes per day - Unsupervised home exercise training included endurance, lower limb, upper limb exercises - Motivational interviewing during weekly phone calls used by physiotherapist to encourage behavior change - Patient selected from menu of educational topics to discuss during weekly phone call with physiotherapist - Weekly phone calls included disease specific self-management training | - Pedometer provided to patients to track walking distance - No special equipment required - Exercise training used daily activities and equipment available in the home |
| Mendes de Oliveira 2010  Brazil | 12 weeks | - In-person group education session - Patients provided with written chart summarizing content covered during education session - Comprehensive assessment conducted at the clinic - Exercise prescription provided - In-person exercise program training at the clinic - Outpatient pulmonary rehabilitation program three times per week for twelve weeks | - In-person group education session - Patients provided with written chart summarizing content covered during education session - Comprehensive assessment conducted at the clinic - Exercise prescription provided - In-person exercise program training at the clinic - Home exercise program three times per week for twelve weeks - Patients recorded exercise activities in a log after each exercise session - Weekly telephone calls from health care provider | - Hand and ankle weights - Heart rate monitor |
| Nolan 2019  UK | 8 weeks | - In-person assessment - Community-based pulmonary rehabilitation comprised of two two-hour sessions per week for eight weeks - Supervised exercise for sixty minutes included endurance and strength training - Educational session were forty-five minutes and covered the same topics as the home-based pulmonary rehabilitation program - Unsupervised exercise sessions at least once per week at home encouraged | - In-person assessment - Individual exercise prescription - Unsupervised exercise for three days per week included endurance and strength training - Weekly telephone calls with physiotherapist - Patients provided with an educational manual | - None required |
| Chaplin 2017  UK | 6-8 weeks | - In-person assessment - Patients were provided with an exercise prescription - Outpatient or Community-based pulmonary rehabilitation comprised of exercise and education sessions - Outpatient program consisted of one-hour twice weekly exercise training sessions supervised for four weeks followed by three weeks unsupervised - Community-based program consisted of one-hour twice weekly exercise training sessions supervised for up to seven - Education sessions for one hour twice weekly for up to seven weeks - Patients were encouraged to exercise at home - Patients were encouraged to complete an exercise diary | - In-person assessment - Patients were provided with an exercise prescription - Patients attended an in-person introductory session to be trained on using the web-based program - Patients received written instructions on how to use the web-based program - Patients were encouraged to exercise daily for six to eight weeks - Patients were encouraged to record their physical activity in the online exercise diary - Patients were contacted by a rehabilitation specialist weekly by email or telephone - The web-based program included educational modules that patients progressed through at their own pace | - Web-based pulmonary rehabilitation program |
| Notes: Usual care: patients were managed by their GP, specialist or both according to local practices. COPD: chronic obstructive pulmonary disease; NR: not reported | | | | |

| Supplementary Appendix 5. Home based pulmonary rehabilitation program components | | | | | | | | |
| --- | --- | --- | --- | --- | --- | --- | --- | --- |
| **Study** | **Program duration** | **Providers delivering program** | **Comprehensive assessment** | **Monitoring** | **Exercise**  **(Types/Total Duration/Total Frequency** | **Education** | **Behaviour change counselling** | **Self-management training** |
| ***HBPR vs usual care*** | | | | | | | | |
| Lahham 2019  Australia | - 8 weeks | - Physiotherapists | - Initial visit 90 minutes in duration - Six-minute walk test used to calculate initial walking speed - Exercise prescription provided | - Home diary provided to patients - Exacerbations recorded in the home diary - Exercise goals and daily exercise recorded in home diary - Pedometer provided to patients - Pedometer used to track daily walking distance - Physiotherapist called the patient once per week - Physiotherapist and patient both recorded weekly exercise goals | - Unsupervised home exercise 5 times per week for 30 minutes per day - Endurance training prescribed as walking 30 - Individual patient goals set for number of repetitions and sets for resistance training - Lower limb resistance training using daily activities and equipment available in the home (step-up using stairs and sit stand from a chair) - Upper limb resistance training using daily activities and equipment in the home (water bottles) | - Patients provided an educational booklet on living with COPD - Home diary included guidelines for identifying and managing exacerbations - Home diary included guidelines for an Action Plan - During weekly phone calls with physiotherapist patients selected topics to discuss from a menu of topics - Menu of optional educational topics included: medication use, managing breathlessness, nutrition, smoking session and community support access | - During weekly phone calls with physiotherapist motivational interviewing used to encourage behavior change through increasing patient confidence, understanding of motivations for change as well as specific goal setting - The physiotherapist used structured telephone modules with prompts to explore and build motivation for change - Physiotherapists completed formal training in motivational interviewing - Physiotherapists were supported throughout the intervention by an expert in motivational interviewing | - During initial in-person home visit, the physiotherapist discussed aspects of self-management that could not be addressed adequately during the subsequent phone calls, such as inhaler technique - Weekly phone calls with physiotherapist included disease specific self-management training - Self-management training focused on advancing disease knowledge, promoting problem solving and facilitating health-related behavior change - Self-management of acute exacerbations was discussed at one session with every patient |
| Coultas 2018  USA | - 26 weeks | - Health coach with undergraduate degree in psychology - 100 hours of in-person and online training on COPD self-management and behavioural change coaching - Reading assignments and role-playing | - Self-management needs assessment - Ten open-ended questions and four sets of structured questions to assess general COPD and physical activity self-management needs - COPD topics assessed included: learning needs, concerns and fears, previous experiences, social support, goals and barriers to self-management - Physical activity assessment included: activities patients enjoy, social supports for physical activity and perceived barriers to physical activity | - Bi-weekly telephone calls with health coach during 20-week unsupervised home exercise program | - 20-week unsupervised home exercise program - 30 minutes of moderate physical activity per day - Bi-weekly phone calls with health coach assists patients to identify enjoyable physical activity such as gardening, walking or dancing - 10-month maintenance program includes monthly automated telephone calls to prompt patients to maintain daily physical activity | - Patients provided with a COPD manual that included the following educational topics: understanding COPD, how to communicate with health care providers, medications, strategies for controlling symptoms, exacerbation management action planning, physical activity, healthy eating, smoking cessation and mental health | - 20-week behavior change program delivered by a health coach using a self-study workbook and weekly telephone calls - Weekly phone calls alternated between one-on-one telephone counselling with a health coach and computer assisted telephone calls - Theoretical foundations of behavior change program were the transtheoretical model (stages of readiness for change) and social cognitive theory (behavior change is based on a complex interaction of intrapersonal, social and environmental factors) - Each week focusses on a different cognitive or behavioural skill such as goal setting, problem solving, identifying benefits of change and cognitive restructuring) - The health coach tailors coaching to appropriate individual stage of change - Coaching and computer assisted calls follow standardized scripts - Ten-month maintenance program consisting of five reading assignments and monthly automated telephone calls - Readings and automated messages focus on self-efficacy | - Patients provided with a COPD self-management manual that included the following self-management topics: how to communicate with health care providers, strategies for controlling symptoms, exacerbation management action plan and mood management - 6-week self-management education program delivered by a health coach using a workbook and weekly telephone calls |
| Li 2018  China | - 12 months | - Not Reported | - Six-minute walk test used to calculate initial walking speed - Exercise prescription provided | - Bi-weekly home visits for two months - Monthly home visit of four months - Weekly telephone calls for six months | - Weekly home exercise program for two months alternating between supervised and unsupervised - Unsupervised weekly home exercise program for ten months - Endurance training graded starting at five minutes per day once a week increasing to twenty minutes per day once a week - Upper limb resistance training once per week - Unsupervised respiratory training for thirty minutes per day three times per week | - Health education delivered in person bi-weekly for two months | - Not Reported | - Not Reported |
| Khoshkesht 2015  Iran | 7 weeks | - Nurses | - Not Reported | - Patients kept daily records on program checklist - Patients recorded respiratory exercises, muscle stretching exercises, episodes of effective cough management, stress reduction methods used, and nutritional recommendations followed - Program checklists were monitored by nurses during weekly phone calls | - Unsupervised muscle stretching exercises three times per week gradually increasing from five minutes per day to twenty minutes per day by adding three minutes each day | - Health education delivered in person in three thirty-minute sessions - Education sessions focused on self-care and included topics such as: nutrition and stress reduction | - Behaviour change was supported through weekly fifteen-minute telephone calls with nurses - Nurses used persuasion techniques to reinforce importance of training - Nurses provided patient with emotional support | - During in-person health education sessions patients were instructed in stress reduction methods such as deep breathing exercises, visual imagery and progressive muscle relaxation - During in-person health education sessions patients were instructed in breath control strategies during exacerbations - During in-person health education sessions the nurses demonstrated the techniques and the patients subsequently performed them in front of the nurses to ensure they were being performed correctly |
| Pradella 2015  Brazil | 8 weeks | - Nurses | - Not Reported | - Patients provided with a log for monitoring activities | - Unsupervised exercise program included warm-up, aerobic activity, stretching and relaxation three times per week - Warm-up included five one-minute exercises - Endurance training graded with a goal of reaching 40 minutes of walking while using pursed lip breathing per day three times a week - Lower limb strength training began in week two with stairs or step-ups while using pursed-lip breathing starting at five minutes per day increasing to fifteen minutes per day three times per week - Upper limb strength training began in week two with 3 sets of 30 repetitions per set three times per week - Stretching of muscle groups in head and neck, upper limbs and lower limbs performed for 20 seconds three times per week - Relaxation exercise consisting of reclining on a pillow listening to music using progressive body part awareness and deep breathing techniques for thirty minutes three times per week | - One week of in-person training at the rehabilitation centre - Patients provided with an educational booklet - Unclear if education included topics beyond exercise | - Behaviour change was supported through weekly telephone calls with nurses | - Unclear if education included topics beyond exercise |
| De Sousa Pinto 2014  Spain | 12 weeks | - Nurses - Physiotherapists | - Assessment of pulmonary function, breathlessness and exercise capacity - Exercise prescription provided | - Home visits to monitor exercise performance and compliance twice per week for two weeks followed by twice per month - Weekly telephone calls to monitor compliance | - Supervised exercise twice per week for two weeks followed by twice per month - Unsupervised exercise weekly (frequency not specified) - Total daily exercise time progressed from twenty minutes to one hour based on individual patient exercise capacity - Endurance training consisted of walking outdoors, cycling, stair climbing or treadmill walking progressing from five minutes per day to thirty minutes per day - Lower limb strength training consisted of two sets with ten repetitions per day and progressively increased weight and number of repetitions - Upper limb strength training consisted of two sets with ten repetitions per day and progressively increased weight and number of repetitions - Stretching and breathing exercises were included in each training session | - In-person session with nurse to receive instruction on inhaler devices and nebulizer therapy - Patients provided with guideline on COPD and COPD treatment - Education provided by physiotherapist in first two in-home visits - Education topics included energy conservation techniques, strategies to cope with symptoms and the role of exercise in managing COPD | - Behaviour change was supported through weekly telephone calls | - Education provided by physiotherapist in first two in-home visits included strategies to cope with symptoms, energy conservation techniques and breathing retraining (diaphragmatic respiration and pursed-lip breathing) |
| Liu 2013  China | 16 weeks | - Nurses | - Exercise capacity assessed using six-minute walk test prior to hospital discharge - Pulmonary function assessed prior to hospital discharge | - System monitored program participation - Nurses contacted patients by telephone if they were not regularly logging into the system - Patients, family members and nurses had access to program participation history to monitor compliance | - Sixteen-week online home based pulmonary rehabilitation program - Guided exercises consisting of fifteen-twenty minutes per session with two sessions per day - Respiratory training included three different types of breathing exercises: pursed-lip breathing, deep inspiration and slow exhalation while making a fist, and deep inspiration with a hold followed by a slow exhalation - Global exercise training included an eight-step upper limb and lower limb sequence | - Unclear if education included topics beyond guided exercise instructions | - Not Reported | - Unclear if self-management training included topics beyond guided exercise instructions |
| Mendes de Oliveira 2010  Brazil | 12 weeks | - Multidisciplinary team of health care providers | - Comprehensive assessment conducted at the clinic - Physical and spirometric evaluation - Six-minute walk test used to calculate initial walking intensity - Exercise prescription provided | - Patients recorded exercise activities in a log after each exercise session - Telephone calls from health care provider (frequency not reported) | - Home exercise program three times per week for twelve weeks - Active warm-up of callisthenic exercises - Endurance training consisted of walking on flat ground for 30 minutes - Upper limb exercises using hand weights (increasing weight every two weeks) while sitting with ten repetitions each of the following exercises: elbow flexion, elbow abduction, shoulder abduction, and shoulder flexion - Lowe limb exercises using ankle weights (increasing weight every two weeks) while sitting with ten repetitions each of the following exercises: hip flexion and knee extension | - In-person group education session covering the following topics: the development and progression of COPD, pharmacological and non-pharmacological treatments, correct use of oxygen, and the importance of following an exercise-based program - Patients provided with written chart summarizing content covered during education session | - Telephone calls from health care provider (frequency not reported) to reinforce importance of exercise program | - Extent of self-management training included in group education session is unclear |
| Moore 2009  UK | 6 weeks | - Physiotherapists | - Patients assessed by a physiotherapist in-person at the clinic - Borg breathlessness scale used to inform exercise prescription | - Patients recorded exercise activities in a diary after each exercise session | - Home exercise program for thirty minutes per day four days per week for six weeks - Patients followed along with an exercise video that included a warm-up, upper limb exercises, lower limb exercises, aerobic exercises and stretching - Warm-up consisted of joint mobilizing and circulatory exercises (shoulder shrugs, marching on the spot) - Endurance training included exercises such as marching on the spot and modified star jumps - Lower limb strength   training included exercises such as squats, sit to stand, and seated knee extensions)   - Upper limb strength training included exercises such as arm raises, wall push offs, and bicep curls - Exercise intensity was self-regulated based on the Borg scale (degree of breathlessness experienced) - Stretching targeting different muscle groups | - Patients provided with an illustrated exercise diary and an educational booklet - Educational booklet included topics such as diagnosing and treating COPD, medications, oxygen therapy, self-management of chest infections and breathlessness, stress and relaxation, exercise and smoking cessation - Patients watched a video on the benefits of exercise with a physiotherapist in-person at the clinic | - Not Reported | - Educational booklet included topics on self-management including: self-management of chest infections and breathlessness, and stress and relaxation |
| Lalmolda 2017  Spain | 8 weeks | - Nurses - Physiotherapists | - Functional exercise and respiratory capacity assessed by physiotherapist - Individualized daily exercise prescription provided to patient by physiotherapist after the first week | - Compliance with daily home exercises reviewed with physiotherapist using a checklist at each supervised exercise session - Ongoing compliance with unsupervised exercise monitored through monthly follow-up telephone calls with nurses for ten months | - Supervised home-based exercise program delivered by physiotherapist for one hour twice a week for eight weeks - Unsupervised daily exercises | - Education on prescribed oral and inhaled medications delivered to patients in person prior to hospital discharge | - Not Reported | - Not Reported |
| ***HBPR vs OPR*** | | | | | | | | |
| Horton 2017  UK | 7 weeks | - Trainee Psychologist - Physiotherapists | - Functional exercise capacity assessed in-person at the hospital - Patients provided with an exercise prescription with initial walking duration and speed informed by exercise capacity tests | - Patients provided with exercise log to track daily physical activity - Telephone calls during week two and week four to monitor patient progress and encourage patients | - Unsupervised exercise program at home with daily endurance training and strength training three times per week for seven weeks - Endurance training involved thirty minutes of walking daily - Exercise intensity was tracked based on the Borg scale (degree of breathlessness experienced) and were increased as Borg scale became lower - Lower limb strength training exercises were performed three times per week in sets of three with eight repetitions and included sit-to-stands and step-ups - Upper limb strength training exercises were performed three times per week in sets of three with eight repetitions and included bicep curls and pull ups | - Introductory educational session in-person at the hospital - Education session focused on readiness to change and self-efficacy - Patients provided with a standardized manual to support a home exercise program | - Education session focused on readiness to change and self-efficacy - Two ten minute motivational telephone calls during week two and week four to provide encouragement | - Education session included self-efficacy training - Patients provided with a standardized manual with tools to support lifestyle change |
| Holland 2017  Australia | - 8 weeks | - Physiotherapists | - Initial visit ninety minutes in duration - Patient assessed to determine if other referrals required - Inhaler technique assessed - Six-minute walk test used to calculate initial walking speed - Exercise prescription provided | - Home diary provided to patients - Exacerbations recorded in the home diary - Exercise goals and daily exercise recorded in home diary - Pedometer provided to patients - Pedometer used to track daily walking distance - Physiotherapist called the patient once per week - Physiotherapist and patient both recorded weekly exercise goals | - Unsupervised home exercise five times per week for thirty minutes per day - Endurance training prescribed as walking thirty - Individual patient goals set for number of repetitions and sets for resistance training - Lower limb resistance training using daily activities and equipment available in the home (step-up using stairs and sit stand from a chair) - Upper limb resistance training using daily activities and equipment in the home (water bottles) | - Patients provided an educational booklet on living with COPD - Home diary included guidelines for identifying and managing exacerbations - Home diary included guidelines for an Action Plan - During weekly phone calls with physiotherapist patients selected topics to discuss from a menu of topics - Menu of optional educational topics included: medication use, managing breathlessness, nutrition, smoking session and community support access | - During weekly phone calls with physiotherapist motivational interviewing used to encourage behavior change through increasing patient confidence, understanding of motivations for change as well as specific goal setting - The physiotherapist used structured telephone modules with prompts to explore and build motivation for change - Physiotherapists completed formal training in motivational interviewing - Physiotherapists were supported throughout the intervention by an expert in motivational interviewing | - During initial in-person home visit, the physiotherapist discussed aspects of self-management that could not be addressed adequately during the subsequent phone calls, such as inhaler technique - Weekly phone calls with physiotherapist included disease specific self-management training - Self-management training focused on advancing disease knowledge, promoting problem solving and facilitating health-related behavior change   Self-management of acute exacerbations was discussed at one session with every patient |
| Mendes de Oliveira 2010  Brazil | 12 weeks | - Multidisciplinary team of health care providers | - Comprehensive assessment conducted at the clinic - Physical and spirometric evaluation - Six-minute walk test used to calculate initial walking intensity - Exercise prescription provided | - Patients recorded exercise activities in a log after each exercise session - Telephone calls from health care provider (frequency not reported) | - Home exercise program three times per week for twelve weeks - Active warm-up of callisthenic exercises - Endurance training consisted of walking on flat ground for thirty minutes - Upper limb exercises using hand weights (increasing weight every two weeks) while sitting with ten repetitions each of the following exercises: elbow flexion, elbow abduction, shoulder abduction, and shoulder flexion - Lowe limb exercises using ankle weights (increasing weight every two weeks) while sitting with ten repetitions each of the following exercises: hip flexion and knee extension | - In-person group education session covering the following topics: the development and progression of COPD, pharmacological and non-pharmacological treatments, correct use of oxygen, and the importance of following an exercise-based program - Patients provided with written chart summarizing content covered during education session | - Telephone calls from health care provider (frequency not reported) to reinforce importance of exercise program | - Extent of self-management training included in group education session is unclear |
| Nolan 2019  UK | 8 weeks | - Respiratory physiotherapists | - In-person assessment - Individual exercise prescription | - Weekly fifteen-minute telephone calls with physiotherapist to review and progress the exercise program, to advise which sections of the educational manual to read and to answer any questions the patient may have regarding the exercises or educational content | - Unsupervised exercise for three days per week included endurance and strength training - Endurance training consisted of walking for thirty minutes per day at least three times per week - Lower limb strength training started at one set with fifteen repetitions and no weight and increased to two sets with twenty repetitions with weight - Upper limb strength training started at one set with fifteen repetitions and no weight and increased to two sets with twenty repetitions with weight - Exercise intensity was graded based on the Borg scale (degree of breathlessness experienced) | - Patients provided with an educational manual that covered the following topics: lung anatomy, COPD pathophysiology, medication, self-management, smoking cessation, mental health, eating and swallowing difficulties, relaxation techniques and management of activities of daily living - Weekly fifteen-minute telephone calls with physiotherapist to advise which sections of the educational manual to read and to answer any questions the patient may have regarding the educational content | - Patients provided with an educational manual that included content on smoking cessation - Weekly fifteen-minute telephone calls with physiotherapist to review and progress the exercise program, to advise which sections of the educational manual to read and to answer any questions the patient may have regarding the exercises or educational content | - Patients provided with an educational manual that covered the following self-management topics: self-management of COPD, identification and management of chest infections, management of anxiety and depression, management of eating and swallowing difficulties, relaxation techniques and management of activities of daily living - Weekly fifteen-minute telephone calls with physiotherapist to review and progress the exercise program, to advise which sections of the educational manual to read and to answer any questions the patient may have regarding the exercises or educational content |
| Chaplin 2017  UK | 6-8 weeks | - Rehabilitation physiotherapists | - In-person assessment of lung function, muscle strength and exercise capacity - Patients were provided with an exercise prescription - Maximal shuttle walking exercise tests used to calculate initial walking intensity | - Patients were encouraged to record their physical activity in the online exercise diary - Patient progress was monitored online by the rehabilitation specialist - Patients were contacted by a rehabilitation specialist weekly by email or telephone | - Patients were encouraged to exercise daily for six to eight weeks - Endurance training consisted of graded walking five days per week - Lower limb strength training with weights three days per week - Upper limb strength training with weights three days per week - Walking time and strength training were progressed to maintain a visual analogue scale rating of four to seven | - The web-based program included educational modules that patients progressed through at their own pace - Education program based on a self-management program of activity, coping and education for COPD - Online modules included reading material on disease education, managing breathlessness, recognizing the signs and symptoms of an exacerbation, energy conservation, diet and healthy eating | - The web-based program included educational modules on smoking cessation - Patients were contacted by a rehabilitation specialist weekly by email or telephone and motivational interviewing techniques were used to promote behavior change | - The web-based program included educational modules that patients progressed through at their own pace - Education program based on a self-management program of activity, coping and education for COPD - Online modules included reading material on managing breathlessness, recognizing the signs and symptoms of an exacerbation, and energy conservation |
| Notes: Usual care: patients were managed by their GP, specialist or both according to local practices.  COPD: chronic obstructive pulmonary disease; HBPR: home-based pulmonary rehabilitation | | | | | | | | |

| Supplementary Appendix 6: Risk of bias in RCTs | | | | | | | | | | | | | | | | | | | | | |
| --- | --- | --- | --- | --- | --- | --- | --- | --- | --- | --- | --- | --- | --- | --- | --- | --- | --- | --- | --- | --- | --- |
| **Study** | **Selection bias** | | | | | **Performance bias** | | | **Detection bias** | | | | | **Attrition bias** | | | **Reporting bias** | | | **Other bias** | |
|  | **Random sequence generation** | **Description** | **Allocation concealment** | **Description** | **Blinding of participants and personnel** | | **Description** | **Blinding of outcome assessment (patient reported outcomes)** | | **Description** | **Blinding of outcome assessment (other outcomes)** | **Description** | **Incomplete outcome data** | | **Description** | **Selective reporting** | | **Description** | **other bias** | | **Description** |
| Lahham 2019  Australia | Low | Computer-generated sequence | Low | Concealed from researchers using an online database | High | | Personnel delivering intervention were not blinded  Participants were not blinded due to the nature of intervention | High | | Participants were not blinded to the intervention | Low | Assessor was blinded to intervention group assignment | High | | Missing data were not balanced between groups | Low | | Trial was registered and pre-specified outcomes were reported in the trial | Low | | Study appears to be free from other sources of bias |
| Coultas 2018  USA | Low | Randomization was conducted by the data coordinating center using a permuted block design | Low | Participants were sequentially assigned unique ID numbers at the time of enrollment  Only study coordinator knew group assignment, which was concealed from other study personnel and patients until after completion of the six-week self management intervention | High | | Personnel delivering intervention were not blinded due to nature of intervention  Participants were not blinded due to the nature of intervention | High | | Participants were not blinded to the intervention | Low | Assessors (interviewers and respiratory therapists) were blinded to intervention group assignment | High | | Factors associated with drop-outs during the intervention period differed between treatment intervention groups  No adjustments were made for missing data | Low | | Protocol was publicly available  All pre-specified outcomes were reported in multiple publications | Low | | Study appears to be free of other sources of bias |
| Horton 2018  UK | Low | Computer generated sequence using a permuted block design | Low | Only researcher uninvolved in the study knew group assignment  Used sealed opaque envelopes | High | | Personnel delivering intervention were not blinded due to nature of intervention  Participants were not blinded due to the nature of intervention | High | | Participants were not blinded to the intervention | Low | Assessors were blinded (outcome measures taken at 7 weeks and 6 months were conducted by a member of staff blinded to the allocation of the participants) | Low | | Undertook modified intention to treat complete case analysis and per protocol analysis  Data from some baseline appointments  and questionnaires were missing  More patients in the home based group had incomplete data, but numbers were still similar between the 2 groups  Imputed values were used to deal with missing data | Unclear | | No trial registration found | Low | | Study appears to be free of other sources of bias |
| Li 2018  China | Unclear | No information provided | Unclear | No information provided | high | | Personnel delivering intervention were not blinded due to nature of intervention  Participants were not blinded due to the nature of intervention | High | | Participants were not blinded to the intervention | Unclear | No information provided | High | | Missing data were not balanced between groups | Unclear | | No trial registration found | Low | | Study appears to be free of other sources of bias |
| Chaplin 2017  UK | Low | Used web-based program | Low | Use web-based central allocation | High | | Personnel delivering intervention were not blinded due to nature of intervention  Participants were not blinded due to the nature of intervention | High | | Participants were not blinded to the intervention | Low | Outcome assessor was blinded to treatment group allocation | High | | Percentage of dropouts was greater in HBPR group than in ‘usual care’ group (57% vs 23%) respectively  Reasons for withdrawal differed between groups | Low | | Trial was registered  Pre-specified outcomes were reported in the study | Low | | Study appears to be free of other sources of bias |
| Holland 2017  Australia | Low | Computer-generated sequence | Low | Used concealed opaque envelopes | High | | Personnel delivering intervention were not blinded due to nature of intervention  Participants were not blinded due to the nature of intervention | High | | Participants were not blinded to the intervention | Low | Outcome assessor was blinded to treatment group allocation | Low | | Reasons for missing data were provided  Reasons were similar between groups | Low | | Trial was registered  Pre-specified outcomes were reported in the study | Low | | Study appears to be free of other sources of bias |
| Khoshkesht 2015  Iran | Unclear | Insufficient information provided | Unclear | No information provided | High | | Personnel delivering intervention were not blinded due to nature of intervention  Participants were not blinded due to the nature of intervention | High | | Participants were not blinded to the intervention | Unclear | No information provided | Low | | Patients with missing data were similar to those with no missing data | Unclear | | No trial registration found | Low | |  |
| Pradella 2015  Brazil | Unclear | No information provided | Unclear | No information provided | High | | Personnel delivering intervention were not blinded due to nature of intervention  Participants were not blinded due to the nature of intervention | High | | Participants were not blinded to the intervention | Unclear | No information provided | Low | | Proportion of missing data was small and similar across groups | Unclear | | No trial registration found | Low | | Study appears to be free of other sources of bias |
| De Sousa Pinto 2014  Spain | Low | Computer-generated sequence | Unclear | Insufficient information provided | High | | Personnel delivering intervention were not blinded due to nature of intervention  Participants were not blinded due to the nature of intervention | High | | Participants were not blinded to the intervention | Unclear | No information provided | Low | | Study did not describe characteristics of patients who did not complete study  Only patients with complete data were included in analyses  Percentage of patients with missing data was small and similar across groups | Unclear | | No trial registration found | Low | | Study appears to be free of other sources of bias |
| Liu 2013  China | Unclear | Insufficient information provided | Low | Used sealed opaque envelopes | High | | Personnel delivering intervention were not blinded due to nature of intervention  Participants were not blinded due to the nature of intervention | High | | Participants were not blinded to the intervention | Low | Assessors (research nurses and investigators) were blinded to treatment group allocation | Low | | Missing outcome data were balanced between groups, with similar reasons for missing data across groups | Unclear | | No trial registration found  . | Low | | Study appears to be free of other sources of bias |
| Mendes de Oliveira 2010  Brazil | Low | Computer-generated sequence | Unclear | No information provided | High | | Personnel delivering intervention were not blinded due to nature of intervention  Participants were not blinded due to the nature of intervention | N/A | | No patient reported outcomes were measured | Unclear | No information provided | High | | Missing data is not balanced between groups. Study only looked at data of patients with no missing data | Unclear | | No trial registration found | Low | | Study appears to be free of other sources of bias |
| Moore 2009  UK | Low | Used a minimization strategy that matched the groups for age (< 65 or > 65 years) and airway obstruction (< FEV_1_ 40% or > FEV_1_ 40%) | Low | Randomization was sent to the chief investigator via sealed envelopes and was not revealed until patients had completed baseline assessment | High | | Personnel delivering intervention were not blinded due to nature of intervention  Participants were not blinded due to the nature of intervention | High | | Participants were not blinded to the intervention | High | Assessors were not blinded to treatment group allocation | Unclear | | Insufficient information provided | Unclear | | No trial registration found | Low | | Study appears to be free of other sources of bias |

| **Supplementary Appendix 7: Risk of bias in non-randomized studies** | | | | | | | | | | | | | | | |
| --- | --- | --- | --- | --- | --- | --- | --- | --- | --- | --- | --- | --- | --- | --- | --- |
| **Study** | **Bias due to confounding** | | **Bias in selection of participants into the study** | | **Bias in measurement of interventions** | | **Bias due to departures from intended interventions** | | **Bias due to missing data** | | **Bias in the measurement of outcomes** | | **Bias in selection of the reported result** | | **OVERALL RISK OF BIAS** |
|  | **Judgement** | **Description** | **Judgement** | **Description** | **Judgement** | **Description** | **Judgement** | **Description** | **Judgement** | **Description** | **Judgement** | **Description** | **Judgement** | **Description** |  |
| Nolan 2019  UK | Moderate | The main confounding variables were measured and adjusted for, but unknown confounding could still be present | Serious | Participants were allowed to choose which intervention they received | Low | Intervention status was well defined and remained the same throughout the study | Moderate | Co-interventions and switch to other interventions were unlikely | Moderate | Reasons for missing data were similar across interventions, but missing data were not addressed in the analysis | No information provided | Insufficient information was provided | Moderate | Protocol was not available, but there was no indication of selective reporting | Serious |
| Lalmolda 2017  Spain | Moderate | The main confounding variables were measured and adjusted for, but unknown confounding could still be present | Low | Most participants eligible for the trial were included  Start of follow-up and start of intervention were the same for all participants | Low | Intervention status was well defined and remained the same throughout the study | Moderate | Co-interventions and switch to other interventions were unlikely. Low adherence could be an issue in the intervention group due to the nature of the intervention. | Moderate | Missing data differed between groups | Low | All outcomes were objective measures | Low | All reported results corresponded to all intended outcomes | Moderate |

| Supplementary Appendix 8. Adverse events and deaths during follow-up period | | | | |
| --- | --- | --- | --- | --- |
| **Study** | **Sample size** | **Adverse events n(%)** | **Deaths due to COPD n(%)** | **Deaths from all causes n(%)** |
| ***HBPR vs usual care*** | | | | |
| Lahham 2019  Australia | HBPR: 29  Usual care: 29 | NR | NR | NR |
| Coultas 2018  USA | HBPR: 149  Usual care: 156 | **18 months:**  HBPR: 60 (40)  Usual care: 65 (42)  p = 0.80 | NR | **18 months:**  HBPR: 4 (3)  Usual care: 4 (3)  p = NR |
| Li 2018  China | HBPR: 82  Usual care: 69 | NR | NR | **At the end of PR (12 months):**  HBPR: 2 (2)  Usual care: 3 (4)  p = NR |
| Khoshkesht 2015  Iran | HBPR: 35  Usual care: 35 | NR | NR | NR |
| Pradella 2015  Brazil | HBPR: 32  Usual care: 18 | NR | NR | **At the end of PR (2 months):**  HBPR: 1 (3)  Usual care: 0 (0)  p = NR |
| De Sousa Pinto 2014  Spain | HBPR: 29  Usual care: 21 | NR | NR | NR |
| Liu 2013  China | HBPR: 30  Usual care: 30 | NR | NR | **At the end of PR (4 months):**  HBPR: 1 (3)  Usual care: 1 (3)  p = NR |
| Mendes de Oliveira 2010  Brazil | HBPR: 42  Usual care: 29 | NR | NR | NR |
| Moore 2009  UK | HBPR: 14  Usual care: 13 | NR | NR | NR |
| Lalmolda 2017  Spain | HBPR: 21  Usual care: 29 | NR | NR | **At 12 months:**  HBPR(n=19): 0 (0)  Usual care: 2 (7)  p=0.51 |
| ***HBPR vs OPR*** | | | | |
| Horton 2017  UK | HBPR: 145  OPR: 142 | HBPR: 14 (5)  OPR: 9 (6)  p = NR | **At the end of PR (7 weeks):**  HBPR: 2 (1)  OPR: 1 (1)  p = NR  **At 6 months:**  HBPR: 2 (1)  OPR: 4 (3)  p = NR | NR |
| Holland 2017  Australia | HBPR: 80  OPR: 86 | HBPR: 0 (0)  OPR: 0 (0)  p = NA | **At the end of PR (2 months):**  HBPR: 1 (1)  OPR: 1 (1)  p = NR  **At 12 months:**  HBPR: 5 (6)  OPR: 4 (5)  p = NR | NR |
| Mendes de Oliveira 2010  Brazil | HBPR: 42  OPR: 46 | NR | NR | NR |
| Nolan 2019  UK | HBPR: 154  OPR: 154 | NR | NR | NR |
| Chaplin 2017  UK | HBPR: 51  OPR: 52 | NR | NR | NR |
| Notes: Usual care: patients were managed by their GP, specialist or both according to local practices.  COPD: chronic obstructive pulmonary disease; HBPR: home-based pulmonary rehabilitation; NR: not reported; OPR: outpatient pulmonary rehabilitation; PR: pulmonary rehabilitation | | | | |
|  |  |  |  |  |

| Supplementary Appendix 9. Health-related quality of life –CAT, AQ 20, or VSRQ | | | | | | | | | | | | |
| --- | --- | --- | --- | --- | --- | --- | --- | --- | --- | --- | --- | --- |
| **Study** | **Sample size** | **PR duration** | **Time** | **CAT** | | | **AQ-20** | | | **VSRQ (total score)** | | |
|  |  |  |  | **HBPR**  **Mean±SD** | **Comparison group**  **Mean±SD** | **Diff. between groups**  **MD(95%CI)** | **HBPR**  **Median (range)** | **Comparison group**  **Median (range)** | **Diff. between groups**  **MD(95%CI)** | **HBPR**  **Mean±SD** | **Comparison group**  **Mean±SD** | **Diff. between groups**  **MD(95%CI)** |
| ***HBPR vs usual care*** | | | | | | | | | | | | |
| Lahham 2019  Australia | HBPR: 29  Usual care: 29 | Active PR phase: 2 months  Maintenance: monthly calls for 6 months | NR | NR | NR | NR | NR | NR | NR | NR | NR | NR |
| Coultas 2018  USA | HBPR: 149  Usual care: 156 | Active PR phase: 6 months  Maintenance: 10 months | NR | NR | NR | NR | NR | NR | NR | NR | NR | NR |
| Li 2018  China | HBPR: 82  Usual care: 69 | Active PR phase: 6 months  Maintenance: weekly calls for 6 months | Baseline | HBPR: 17.6±7.1 | Usual care: 15.9±7.9 | NR | NR | NR | NR | NR | NR | NR |
|  |  |  | 3 months  *Diff. within groups from baseline* | HBPR(n=76): 12.8±6.8  p <0.001 | Usual care(n=62): 13.0±7.0  p <0.001 | p>0.05 | NR | NR | NR | NR | NR | NR |
|  |  |  | 6 months  *Diff. within groups from baseline* | HBPR(n=71): 9.9±5.6  p <0.001 | Usual care (n=55): 11.5±6.2  p <0.001 | p>0.05 | NR | NR | NR | NR | NR | NR |
|  |  |  | 9 months  *Diff. within groups from baseline* | HBPR(n=69): 8.5±5.0  p <0.001 | Usual care: (48): 13.2±6.1  p <0.001 | P<0.001 | NR | NR | NR | NR | NR | NR |
|  |  |  | 12 months  *Diff. within groups from baseline* | HBPR(n=61): 8.5±4.9  p <0.001 | Usual care (n=43): 15.1±6.2  p =0.91 | P<0.001 | NR | NR | NR | NR | NR | NR |
| Khoshkesht 2015  Iran | HBPR: 35  Usual care: 35 | Active PR phase: 2 months | NR | NR | NR | NR | NR | NR | NR | NR | NR | NR |
| Pradella 2015  Brazil | HBPR: 32  Usual care: 18 | Active PR phase: 2 months | NR | NR | NR | NR | NR | NR | NR | NR | NR | NR |
| De Sousa Pinto 2014  Spain | HBPR: 29  Usual care: 21 | Active PR phase: 3 months | NR | NR | NR | NR | NR | NR | NR | NR | NR | NR |
| Liu 2013  China | HBPR: 30  Usual care: 30 | Active PR phase: 4 months | NR | NR | NR | NR | NR | NR | NR | NR | NR | NR |
| Mendes de Oliveira 2010  Brazil | HBPR: 42  Usual care: 29 | Active PR phase: 3 months | NR | NR | NR | NR | NR | NR | NR | NR | NR | NR |
| Moore 2009  UK | HBPR: 14  Usual care: 13 | Active PR phase: 1.5 months | NR | NR | NR | NR | NR | NR | NR | NR | NR | NR |
| Lalmolda 2017  Spain | HBPR: 21  Usual care: 29 | Active PR phase: 2 months  Maintenance: monthly calls for 12 months | NR | NR | NR | NR | NR | NR | NR | NR | NR | NR |
| ***HBPR vs OPR*** | | | | | | | | | | | | |
| Horton 2017  UK | HBPR: 145  OPR: 142 | Active PR phase: 2 months | NR | NR | NR | NR | NR | NR | NR | NR | NR | NR |
| Holland 2017  Australia | HBPR: 80  OPR: 86 | Active PR phase: 2 months | NR | NR | NR | NR | NR | NR | NR | NR | NR | NR |
| Mendes de Oliveira 2010  Brazil | HBPR: 42  OPR: 46 | Active PR phase: 3 months | NR | NR | NR | NR | NR | NR | NR | NR | NR | NR |
| Nolan 2019  UK | HBPR: 154  OPR: 154 | Active PR phase: 2 months | NR | NR | NR | NR | NR | NR | NR | NR | NR | NR |
| Chaplin 2017  UK | HBPR: 51  OPR: 52 | Active PR phase: around 3 months | Baseline | HBPR: 20.8±8.6 | OPR: 20.8±7.5 | NR | NR | NR | NR | NR | NR | NR |
|  |  |  | At PR completion | HBPR:NR | OPR: NR | p >0.05 | NR | NR | NR | NR | NR | NR |
| *Difference between non-socially deprived and socially deprived groups.  Notes: Usual care: patients were managed by their GP, specialist or both according to local practices. AQ-20: airway questionnaire 20; CAT: COPD assessment test; CI: Confidence interval; COPD: chronic obstructive pulmonary disease; HBPR: home-based pulmonary rehabilitation; MD: Mean difference; NA: not applicable; NR: not reported; OPR: outpatient pulmonary rehabilitation; PR: pulmonary rehabilitation; SD: standard deviation; ; SGRQ: St. George’s respiratory questionnaire; VSRQ: visual simplified respiratory questionnaire | | | | | | | | | | | | |

| Supplementary Appendix 10. Health related quality of life - Chronic Respiratory Disease Questionnaire (CRQ) | | | | | | | | | | | | | | | |
| --- | --- | --- | --- | --- | --- | --- | --- | --- | --- | --- | --- | --- | --- | --- | --- |
| **Study** | **Sample size** | **PR duration** | **Time** | **Dyspnoea** | | | **Emotional function** | | | **Fatigue** | | | **Mastery** | | |
|  |  |  |  | **HBPR**  **Mean±SD**  **Median(IQR)** | **Comparison group**  **Mean±SD**  **Median(IQR)** | **Diff. between groups**  **MD(95%CI)** | **HBPR**  **Mean±SD**  **Median(IQR)** | **Comparison group**  **Mean±SD**  **Median(IQR)** | **Diff. between groups**  **MD(95%CI)** | **HBPR**  **Mean±SD**  **Median(IQR)** | **Comparison group**  **Mean±SD**  **Median(IQR)** | **Diff. between groups**  **MD(95%CI)** | **HBPR**  **Mean±SD**  **Median(IQR)** | **Comparison group**  **Mean±SD**  **Median(IQR)** | **Diff. groups**  **MD(95%CI)** |
| ***HBPR vs no usual care*** | | | | | | | | | | | | | | | |
| Lahham 2019  Australia | HBPR: 29  Usual care: 29 | Active PR phase: 2 months  Maintenance: monthly calls for 6 months | Baseline | 19±7 | 19±5 | NR | 34±8 | 37±7 | NR | 17±6 | 17±5 | NR | Median (IQR)  24(18-27) | 23(19-25) | NR |
|  |  |  | 2 months  *Diff. within group*  *MD(95%CI)* | NR  *2.6 (-0.9, 5.8)*  p >0.05 | NR  *2.2 (1.1, 5.6)*  p >0.05 | 1.0 (-2.4, 4.4)  p >0.05 | NR  *2.6 (-1.6, 6.9)*  p >0.05 | NR  *-0.6 (-4.8, 5.6)*  p >0.05 | 0.6 (-3.7, 4.9)  p >0.05 | NR  *3.8 (1.0, 6.5)*  p <0.05 | NR  *1.0 (-1.7, 3.7)*  p >0.05 | 3.4 (0.6, 6.2)  p <0.05 | NR  *2.3 (-0.2, 4.8)*  p >0.05 | NR  *2.0 (-0.5, 4.5)* p >0.05 | 0.3 (-2.2, 2.8) p >0.05 |
|  |  |  | 6 months  *Diff. within group*  *MD(95%CI)* | NR  *3.7(-0.2, 6.5)*  p >0.05 | NR  *2.1 (-1.4, 5.7)*  p > 0.05 | 1.7(-2.0, 5.4)  p >0.05 | 3.5(-0.9, 7.9)  p >0.05 | NR  *-0.6 (-5.1, 5.9)*  p >0.05 | 1.4(-3.3, 6.1)  p >0.05 | NR  *2.8 (0.0, 5.6)* p <0.05 | NR  *1.4 (-1.4, 4.3)*  p >0.05 | 1.9(-1.1, 5.0)  p >0.05 | NR  *1.6 (-0.9, 4.2)*  p >0.05 | NR  *0.9 (-1.7, 3.6)* p >0.05 | 0.7(-2.1, 3.5) p >0.05 |
| Coultas 2018  USA | HBPR: 149  Usual care: 156 | Active PR phase: 6 months  Maintenance: 10 months | Baseline | 4.5±1.3 | 4.3±1.3 | NR | NR | NR | NR | NR | NR | NR | NR | NR | NR |
|  |  |  | 18 months  *Diff. within group*  *MD(95%CI)* | n=113  4.5±1.4  *-0.03 (NR)*  p >0.05 | n=134  4.23±1.49  *-0.09 (NR)*  p >0.05 | NR  p = 0.18 | NR | NR | NR | NR | NR | NR | NR | NR | NR |
| Li 2018  China | Usual care: 82  Usual care: 69 | Active PR phase: 6 months  Maintenance: weekly calls for 6 months | NR | NR | NR | NR | NR | NR | NR | NR | NR | NR | NR | NR | NR |
| Khoshkesht 2015  Iran | HBPR: 35  Usual care: 35 | Active PR phase: 2 months | NR | NR | NR | NR | NR | NR | NR | NR | NR | NR | NR | NR | NR |
| Pradella 2015  Brazil | HBPR: 32  Usual care: 18 | Active PR phase: 2 months | NR | NR | NR | NR | NR | NR | NR | NR | NR | NR | NR | NR | NR |
| De Sousa Pinto 2014  Spain | HBPR: 29  Usual care: 21 | Active PR phase: 3 months | NR | NR | NR | NR | NR | NR | NR | NR | NR | NR | NR | NR | NR |
| Liu 2013  China | HBPR: 30  Usual care: 30 | Active PR phase: 4 months | NR | NR | NR | NR | NR | NR | NR | NR | NR | NR | NR | NR | NR |
| Mendes de Oliveira 2010  Brazil | HBPR: 42  Usual care: 29 | Active PR phase: 3 months | NR | NR | NR | NR | NR | NR | NR | NR | NR | NR | NR | NR | NR |
| Moore 2009  UK | HBPR: 14  Usual care: 13 | Active PR phase: 1.5 months | Baseline | n=10: 3.3 (1.8-4.1) | n=10: 2.7 (2.0-4.8) | NR | n=10: 4.4 (3.2–5) | n=10: 4 (2.4–5.8) | NR | n=10: 2.9 (2–4.4) | n=10: 2.5 (2–4.6) | NR | n=10: 4.8 (4.1–5.5) | n=10: 3.5 (2.9–5.6) | NR |
|  |  |  | 1.5 months  *Diff. within group*  *Median(IQR)* | n=10:  3.6 (2.6–4.4)  *0.5 (0.2–0.6)*  *p* = 0.03 | n=10: 2.5 (2.0–3.2)  *–0.1(–1.5–0.3)*  *p* = 0.33 | p =0.04 | n=10:  5.4 (4.8–6.0)  *0.8 (0.6–2.1)*  *p <0.01* | n=10: 4 (2.9–5.9)  *0.2 (-0.3-0.5)*  *p* = 0.73 | p <0.001 | n=10:  4.9 (4–5.1)  *1.7 (0.6–2.4)*  *p <0.01* | n=10:  2.5 (1.9–4.5)  *0.0 (-1.4-0.7)*  *p* = 0.74 | p = 0.01 | n=10: 5.4 (4.6–5.9)  *0.6 (–0.1–1.4)*  *p* = 0.06 | n=10: 4.5 (3–5.4)  *0.8 (–0.9-1.1)*  *p* = 0.64 | p = 0.25 |
| Lalmolda 2017  Spain | HBPR: 21  Usual care: 29 | Active PR phase: 2 months  Maintenance: monthly calls for 12 months | NR | NR | NR | NR | NR | NR | NR | NR | NR | NR | NR | NR | NR |
| ***HBPR vs OPR*** | | | | | | | | | | | | | | | |
| Horton 2017  UK | HBPR: 145  OPR: 142 | Active PR phase: 2 months | Baseline | HBPR(n=137): 2.6±0.9 | OPR(n=129): 2.4±0.9 | NR | HBPR(n=137): 4.4±1.2 | OPR(n=129): 4.4±1.2 | NR | HBPR(n=137): 3.4±1.2 | OPR(n=129): 3.4±1.2 | NR | HBPR(n=137): 4.5±1.4 | OPR (n=129): 4.4±1.3 | NR |
|  |  |  | 2 months | HBPR (n=79): 3.1±1.3 | OPR(n=83): 3.4±1.2 | −0.2(−0.6, 0.1)  p = 0.18 | HBPR(n=82): 4.5±1.20 | OPR(n=81): 4.9±1.1 | −0.4 (−0.7, 0.2)  p = 0.01 | HBPR(n=82): 3.7±1.2 | OPR(n=83): 4.1±1.5 | −0.4 (−0.7, 0.1)  p = 0.02 | HBPR(n=82): 4.8±1.3 | OPR(n=82): 5.0±1.2 | -0.4 (-0.7, 0.1)  p = 0.02 |
|  |  |  | 6 months | HBPR (n=66): 2.8±1.2 | OPR(n=70): 3.1±1.3 | −0.4 (−0.7, 0.03)  p = 0.07 | HBPR(n=67): 4.3±1.3 | OPR(n=71): 4.5±1.2 | −0.3 (−0.6, 0.1)  p = 0.09 | HBPR(n=67): 3.5±1.4 | OPR(n=71): 3.6±1.3 | −0.2 (−0.6, 0.2)  p = 0.38 | HBPR(n=67): 4.5±1.5 | OPR(n=71): 4.6±1.4 | −0.3(−0.7, 0.1)  p = 0.14 |
| Holland 2017  Australia | HBPR: 80  OPR: 86 | Active PR phase: 2 months | Baseline | HBPR: NR | OPR: NR | NR | HBPR: NR | OPR: NR | NR | HBPR: NR | OPR: NR | NR | HBPR: NR | OPR: NR | NR |
|  |  |  | 2 months  *Diff. within group*  *MD(95%CI)* | HBPR(n=72): NR  *4.4(2.9, 5.6)*  p = NR | OPR(n=76): NR  *2.7(1.4, 4.0)*  p = NR | 1.6 (-0.3, 3.4)  p = NR | HBPR(n=72): NR  *3.1(1.5, 4.8)*  p = NR | OPR(n=76): NR  *2.3(0.7, 3.9)*  p = NR | 0.8 (-1.5, 3.1)  p = NR | HBPR(n=72): NR  *2.2(1.1, 3.2)*  p = NR | OPR(n=76): NR  *1.3(0.3, 2.4)*  p = NR | 0.8 (-0.6, 2.3)  p=NR | HBPR(n=72): NR  *2.4(1.4, 3.5)*  p = NR | OPR(n=76): NR  *2.0(1.0, 3.0)*  p = NR | 0.4 (-1.0, 1.8)  p = NR |
|  |  |  | 12 months  *Diff. within group*  *MD(95%CI)* | HBPR(n=72): NR  *1.9(0.5, 3.4)*  p = NR | OPR(n=76): NR  *1.9(0.5, 3.3)*  p = NR | 0.0 (-2.0, 2.1)  p = NR | HBPR(n=72): NR  *3. (1.3, 4.8)*  p = NR | OPR(n=76): NR  *2.6(0.8, 4.3)*  p = NR | 0.5 (-2.0, 3.0)  p = NR | HBPR(n=72): NR  *0.9(-0.2, 2.0)*  p = NR | OPR(n=76): NR  *1.2(0.1, 2.3)*  p = NR | -0.3 (-1.9, 1.3)  p = NR | HBPR(n=72): NR  *2.1(1.0, 3.2)*  p = NR | OPR(n=76): NR  *1.4(0.3, 2.6)*  p =NR | 0.7 (-0.9, 2.2)  p=NR |
| Mendes de Oliveira 2010  Brazil | HBPR: 42  OPR: 46 | Active PR phase: 3 months | NR | NR | NR | NR | NR | NR | NR | NR | NR | NR | NR | NR | NR |
| Nolan 2019  UK | HBPR: 154  OPR: 154 | Active PR phase: 2 months | Baseline | HBPR (n=154): 13.2±5.7 | OPR (n=154): 13.1±5.8 | p=0.45 | HBPR (n=154): 28.9±9.8 | OPR (n=154): 29.2±10.1 | p=0.58 | HBPR (n=154): 12.4±5.4 | OPR (n=154): 13.3±5.2 | p=0.30 | HBPR (n=154): 17.1±5.8 | OPR (n=154): 16.3±5.7 | p=0.74 |
|  |  |  | 2 months  *Diff. within group*  *MD(95%CI)* | HBPR(n=86): NR  *3.8(2.5, 5.2)*  p = NR | OPR(n=98): NR  *5.0(3.6, 6.4)*  p = NR | -1.2 (-3.1, 0.7)  p = 0.23 | HBPR(n=86): NR  *4.1(2.4, 5.8)*  p = NR | OPR(n=98): NR  *4.4(2.8,6.0)*  p = NR | -0.3 (-2.6, 2.0)  p = 0.81 | HBPR(n=86): NR  *2.7(1.8, 3.6)*  p = NR | OPR(n=98): NR  *2.9(2.1, 3.8)*  p = NR | -0.3 (-1.5, 1.0)  p = 0.67 | HBPR(n=86): NR  *2.4(1.3, 3.6)*  p = NR | OPR(n=98): NR  *3.4(2.3, 4.4)*  p = NR | -0.9 (-2.5, 0.6)  p = 0.23 |
| Chaplin 2017  UK | HBPR: 51  OPR: 52 | Active PR phase: around 3 months | Baseline | HBPR: 2.7±1.2 | OPR: 2.7±1.1 | NR | NR | NR | NR | NR | NR | NR | NR | NR | NR |
|  |  |  | Program completion  *Diff. within group*  *MD±SD* | HBPR: NR  *0.7±.12*  p <0.001 | OPR: NR  *0.8±1.0*  p <0.001 | p>0.05 | NR | NR | NR | NR | NR | NR | NR | NR | NR |
| Notes: Usual care: patients were managed by their GP, specialist or both according to local practices. CI: Confidence interval; COPD: chronic obstructive pulmonary disease; HBPR: home-based pulmonary rehabilitation; IQR: interquartile range; MD: Mean difference; NR: not reported; OPR: outpatient pulmonary rehabilitation; PR: pulmonary rehabilitation; SD: standard deviation | | | | | | | | | | | | | | | |

| Supplementary Appendix 11. Health related quality of life – St George’s Respiratory Questionnaire | | | | | | | | | | | | | | | |
| --- | --- | --- | --- | --- | --- | --- | --- | --- | --- | --- | --- | --- | --- | --- | --- |
| **Study** | **Sample size** | **PR duration** | **Time** | **Symptoms** | | | **Activity** | | | **Impact** | | | **Total score** | | |
|  |  |  |  | **HBPR**  **Mean±SD** | **Comparison group**  **Mean±SD** | **Diff. between groups**  **MD(95%CI)** | **HBPR**  **Mean±SD** | **Comparison group**  **Mean±SD** | **Diff. between groups**  **MD(95%CI)** | **HBPR**  **Mean±SD** | **Comparison group**  **Mean±SD** | **Diff. between groups**  **MD(95%CI)** | **HBPR**  **Mean±SD** | **Comparison group**  **Mean±SD** | **Diff. between groups**  **MD(95%CI)** |
| ***HBPR vs usual care*** | | | | | | | | | | | | | | | |
| Lahham 2019  Australia | HBPR: 29  Usual care: 29 | Active PR phase: 2 months  Maintenance: monthly calls for 6 months | NR | NR | NR | NR | NR | NR | NR | NR | NR | NR | NR | NR | NR |
| Coultas 2018  USA | HBPR: 149  Usual care: 156 | Active PR phase: 6 months  Maintenance: 10 months | NR | NR | NR | NR | NR | NR | NR | NR | NR | NR | NR | NR | NR |
| Li 2018  China | HBPR: 82  Usual care: 69 | Active PR phase: 6 months  Maintenance: weekly calls for 6 months | NR | NR | NR | NR | NR | NR | NR | NR | NR | NR | NR | NR | NR |
| Khoshkesht 2015  Iran | HBPR: 35  Usual care: 35 | Active PR phase: 2 months | NR | NR | NR | NR | NR | NR | NR | NR | NR | NR | NR | NR | NR |
| Pradella 2015  Brazil | HBPR: 32  Usual care: 18 | Active PR phase: 2 months | Baseline | HBPR (n=29): 57.0+19.4 | Usual care (n=29): 50.8+18.9 | NR | HBPR (n=29): 54.2+25.3 | Usual care (n=29): 63.1+23.1 | NR | HBPR (n=29): 47.3+24.8 | Usual care (n=29): 43.8+29.3 | NR | HBPR (n=29): 50.3+20.9 | Usual care (n=29): 49.1+23.2 | NR |
|  |  |  | 2 months  *Diff. within groups*  *Mean±SD* | HBPR(n=29): 47.9+18.9  *-9.1±21.0*  p >0.05 | Usual care (n=29): 47.7+22.1  *-3.1±21.0*  p >0.05 | 6.0 (-19.4, 7.4)  p >0.05 | HBPR(n=29): 46.1+25.1  *-8.1±24.1*  p >0.05 | Usual care (n=29): 66.3+24.9  *54.2±25.3*  p >0.05 | 11.4(-25.3, 2.5)  p >0.05 | HBPR(n=29): 43.3+23.2  *-4.0±20.6*  p >0.05 | Usual care (n=29): 48.5+30.2  *4.7±12.3*  p >0.05 | 8.7 (-20.4 to 3.0)  p >0.05 | HBPR(n=29): 43.6+18.5  *-6.4±16.1*  p <0.05 | Usual care (n=29): 52.3±24.5  *3.1±12.2*  p >0.05 | 9.7 (-1.0 to -0.1)  *p* <0.05 |
| De Sousa Pinto 2014  Spain | HBPR: 29  Usual care: 21 | Active PR phase: 3 months | Baseline | HBPR (n=23): 55.8±19.0 | Usual care (n=18): 63.2±15.8 | p >0.05 | HBPR (n=23): 74.3±15.0 | Usual care (n=18): 81.5±13.8 | p >0.05 | HBPR (n=23): 46.8±13.6 | Usual care (n=18): 51.6±15.4 | p >0.05 | HBPR (n=23): 56.3±13.1 | Usual care (n=18): 62.3±11.6 | p >0.05 |
|  |  |  | 3 months  *Diff. within groups* | HBPR(n=23): 52.6±21.4  p >0.05 | Usual care (n=18): 62.1±22.0  p >0.05 | p >0.05 | HBPR (n=23): 65.2±17.2  p = 0.01 | Usual care (n=18): 81.8±17.0  p >0.05 | P<0.01 | HBPR(n=23): 33.4±12.8  p <0.001 | Usual care (n=18): 58.0±10.1  p = 0.012 | p <0.001 | HBPR(n=23): 46.0±12.0  p <0.001 | Usual care (n=18): 65.6±10.6  p = 0.03 | p <0.001 |
| Liu 2013  China | HBPR: 30  Usual care: 30 | Active PR phase: 4 months | Baseline | HBPR(n=29): 62.6±0.2 | Usual care (n=28): 62.0±0.3 | p = 0.06 | HBPR(n=29): 52.0±0.3 | Usual care (n=28): 52.6±0.3 | p=0.16 | HBPR(n=29): 46.0±0.3 | Usual care (n=28): 44.7±0.6 | p=0.08 | HBPR(n=29): 53.9±0.3 | Usual care (n=28): 54.5±0.2 | 0.10 |
|  |  |  | 4 months  *Diff. within groups* | HBPR(n=29): 42.0±0.3  p <0.05 | Usual care (n=28): 59.6±1.2  p >0.05 | NR | HBPR(n=29): 34.5±0.3  p <0.05 | Usual care (n=28): 51.0±1.0  p >0.05 | NR | HBPR(n=29): 27.1±0.3  p <0.05 | Usual care (n=28): 41.4±0.8  p >0.05 | NR | HBPR(n=29): 35.3±0.3  p <0.05 | Usual care (n=28): 53.8±1.0  p >0.05 | NR |
|  |  |  |  |  |  |  |  |  |  |  |  |  |  |  |  |
|  |  |  |  |  |  |  |  |  |  |  |  |  |  |  |  |
| Mendes de Oliveira 2010  Brazil | HBPR: 42  Usual care: 29 | Active PR phase: 3 months | NR | NR | NR | NR | NR | NR | NR | NR | NR | NR | NR | NR | NR |
| Moore 2009  UK | HBPR: 14  Usual care: 13 | Active PR phase: 1.5 months | NR | NR | NR | NR | NR | NR | NR | NR | NR | NR | NR | NR | NR |
| Lalmolda 2017  Spain | HBPR: 21  Usual care: 29 | Active PR phase: 2 months  Maintenance: monthly calls for 12 months | NR | NR | NR | NR | NR | NR | NR | NR | NR | NR | NR | NR | NR |
| ***HBPR vs OPR*** | | | | | | | | | | | | | | | |
| Horton 2017  UK | HBPR: 145  OPR: 142 | Active PR phase: 2 months | NR | NR | NR | NR | NR | NR | NR | NR | NR | NR | NR | NR | NR |
| Holland 2017  Australia | HBPR: 80  OPR: 86 | Active PR phase: 2 months | NR | NR | NR | NR | NR | NR | NR | NR | NR | NR | NR | NR | NR |
| Mendes de Oliveira 2010  Brazil | HBPR: 42  OPR: 46 | Active PR phase: 3 months | NR | NR | NR | NR | NR | NR | NR | NR | NR | NR | NR | NR | NR |
| Nolan 2019  UK | HBPR: 154  OPR: 154 | Active PR phase: 2 months | NR | NR | NR | NR | NR | NR | NR | NR | NR | NR | NR | NR | NR |
| Chaplin 2017  UK | HBPR: 51  OPR: 52 | Active PR phase: around 3 months | NR | NR | NR | NR | NR | NR | NR | NR | NR | NR | NR | NR | NR |
| Notes: Usual care: patients were managed by their GP, specialist or both according to local practices. CI: Confidence interval; COPD: chronic obstructive pulmonary diseaseHBPR: home-based pulmonary rehabilitationMD: Mean difference; NR: not reported; OPR: outpatient pulmonary rehabilitation; PR: pulmonary rehabilitation; SD: standard deviation; SGRQ: St. George’s respiratory questionnaire | | | | | | | | | | | | | | | |

| Supplementary Appendix 12. Patient adherence to/compliance with HBPR or comparator | | | |
| --- | --- | --- | --- |
| **Study** | **Sample size** | **Completion of PR follow-up (adherence)**  **n(%)** | **Compliance with PR as defined by study**  **n(%)** |
| ***HBPR vs usual care*** | | | |
| Lahham 2019  Australia | HBPR: 29  Usual care: 29 | HBPR: 27 (93)  Dropouts: 2  Median (range) number of attended sessions: 7 (3-8)  Usual care: 25 (86)  Dropouts: 4  p = NR | NR |
| Coultas 2018  USA | HBPR: 149  Usual care: 156 | NR | NR |
| Li 2018  China | HBPR: 82  Usual care: 69 | HBPR: 68 (83)  Dropouts: 5  Exacerbation: 7  Death: 2  Usual care: 43 (62)  Dropouts:7  Exacerbation: 16  Death: 3  p = NR | *Completion of >50% of scheduled training sessions*  HBPR: 61 (74)  Usual care: NA |
| Khoshkesht 2015  Iran | HBPR: 35  Usual care: 35 | HBPR: 34 (97)  Dropouts: 1  Usual care: 32 (91)  Dropouts: 3  p = NR | NR |
| Pradella 2015  Brazil | HBPR: 32  Usual care: 18 | HBPR: 29 (91)  Dropouts: 1  Exacerbation: 1  Death: 1  Usual care: 15 (83)  Dropout: 2  Exacerbation: 1  p = NR | NR |
| De Sousa Pinto 2014  Spain | HBPR: 29  Usual care: 21 | HBPR: 23 (79)  Lost to follow-up: 6  Usual care: 18 (86)  Lost to follow-up: 3  p = NR | NR |
| Liu 2013  China | HBPR: 30  Usual care: 30 | HBPR: 29 (97)  Death: 1  Usual care: 28 (93)  Exacerbation: 1  Death: 1  p = NR | *Regular breathing exercises*:  HBPR*: 25 (83)  Usual care*: 14 (47)  p=NR |
| Mendes de Oliveira 2010  Brazil | HBPR: 42  Usual care: 29 | HBPR: 33 (79)  Dropouts: 7  Lost to follow-up: 2  Usual care: 29 (100)  p = NR | NR |
| Moore 2009  UK | HBPR: 14  Usual care: 13 | HBPR: 10 (71)  Exacerbation: 3  Knee pain: 1  Usual care: 10 (77)  Lost to follow up: 2  Exacerbation: 1  p = NR | NR |
| Lalmolda 2017  Spain | HBPR: 21  Usual care: 29 | HBPR: 19 (90)  Usual care: 29 (100)  p = NR | NR |
| ***HBPR vs OPR*** | | | |
| Horton 2017  UK | HBPR: 145  OPR: 142 | HBPR: 94 (65)  Dropouts: 31  Death: 2  Comorbidities: 16  Wanted OPR: 2  OPR: 84 (59)  Dropouts: 42  Death: 1  Comorbidities: 12  Wanted HBPR: 3  p = NR | NR |
| Holland 2017  Australia | HBPR: 80  OPR: 86 | HBPR: 73 (91)  Dropouts: 5  Lost to follow-up: 1  Death: 1  OPR: 77 (90)  Dropouts: 7  Lost to follow-up: 1  Death: 1  p = NR | *Completion of >70% of planned sessions*  HBPR: 73 (91)  Dropouts: 5  Unwell: 1  Death: 1  OPR: 42 (49)  Dropouts: 16  Unwell: 14  Pain: 4  Travel limitations: 3  Mental health: 3  Other commitments: 2  Wanted HBPR: 2  p=NR |
| Mendes de Oliveira 2010  Brazil | HBPR: 42  OPR: 46 | HBPR: 33 (79)  Dropouts: 7  Lost to follow-up: 2  OPR: 23 (50)  Dropouts: 19  Lost to follow-up: 4  p = NR | NR |
| Nolan 2019  UK | HBPR: 154  OPR: 154 | HBPR: 86 (56)  OPR: 98 (64)  p = NR | NR |
| Chaplin 2017  UK | HBPR: 51  OPR: 52 | HBPR: 22 (43)  Lost to follow-up: 4  Comorbidities: 5  COPD ill health: 5  Preferences for PR classes: 3  Time: 5  Could not engage with website: 3  Not suitable for home exercise: 2  Broken computer: 1  Social/ family reasons: 1  OPR: 40 (77)  Lost to follow-up: 6  Comorbidities: 2  Preferences for HBPR: 1  Time: 1  Does not want to exercise: 1  Social/ family reasons: 1  p = NR | NR |
| *Regular breathing exercises were measured in different ways across groups. Measure was based on online records for HBPR and by self-report on Usual care patients.  Notes: Usual care: patients were managed by their GP, specialist or both according to local practices.  COPD: chronic obstructive pulmonary disease; HBPR: home-based pulmonary rehabilitation; NA: not applicable; NR: not reported; OPR: outpatient pulmonary rehabilitation; PR: pulmonary rehabilitation | | | |

| Supplementary Appendix 13. Frequency of exacerbations, hospital admissions and ER visits | | | | | | |
| --- | --- | --- | --- | --- | --- | --- |
| **Study** | **Sample size** | **Exacerbations**  **n(%)** | **Hospital admission due to COPD**  **n(%)** | **Hospital admission**  **(all causes)**  **n(%)** | **Length of hospitalization in days**  **Median (IQR)** | **ER visits**  **n(%)** |
| ***HBPR vs usual care*** | | | | | | |
| Lahham 2019  Australia | HBPR: 29  Usual care: 29 | NR | NR | NR |  | NR |
| Coultas 2018  USA | HBPR: 149  Usual care: 156 | NR | **18 months**  HBPR: 28 (19)  Usual care: 47 (30)  p = NR | **18 months**  HBPR: 62 (42)  Usual care: 68 (44)  p = NR | NR | NR |
| Li 2018  China | HBPR: 82  Usual care: 69 | **At the end of PR (12 months):**  HBPR: 7 (9)  Usual care: 16 (23)  p = 0.02 | NR | NR | NR | NR |
| Khoshkesht 2015  Iran | HBPR: 35  Usual care: 35 | NR | NR | NR | NR | NR |
| Pradella 2015  Brazil | HBPR: 32  Usual care: 18 | NR | NR | NR | NR | NR |
| De Sousa Pinto 2014  Spain | HBPR: 29  Usual care: 21 | NR | NR | NR | NR | NR |
| Liu 2013  China | HBPR: 30  Usual care: 30 | **At the end of PR (4 months):**  HBPR: NR  Usual care: 1 (3)  p = NR | NR | NR | NR | NR |
| Mendes de Oliveira 2010  Brazil | HBPR: 42  Usual care: 29 | NR | NR | NR | NR | NR |
| Moore 2009  UK | HBPR: 14  Usual care: 13 | **At the end of PR (2 months):**  HBPR: 3 (21)  Usual care: 1 (8)  p = NR | NR | NR | NR | NR |
| Lalmolda 2017  Spain | HBPR: 21  Usual care: 29 | **At the end of PR (2 months):**  HBPR (n=19): 2 (11)  Usual care: 8 (28)  p = 0.16  **At 12 months:**  HBPR (n=19): 8 (42)  Usual care: 25 (86)  p = NR | **At the end of PR (2 months):**  HBPR (n=19): 2 (11)  Usual care: 4 (14)  **At 12 months:**  HBPR (n=19): 7 (37)  Usual care: 19 (66)  p_adj_= 0.01  p_ITT_= 0.26 | NR | **At the end of PR (2 months):**  HBPR (n=19): 0 (0-0)  Usual care: 0 (0-0)  p = 0.59  **At 12 months:**  HBPR (n=19): 0 (0-7)  Usual care: 7 (0-12)  p =0.03  p_ITT_ = 0.12 | NR |
| ***HBPR vs OPR*** | | | | | | |
| Horton 2017  UK | HBPR: 145  OPR: 142 | NR | **At the end of PR (7 weeks)**:  HBPR: 8 (6)  OPR: 3 (2)  p = NR | **At the end of PR (7 weeks)**:  HBPR: 4 (3)  OPR: 3 (2)  p = NR | NR | NR |
| Holland 2017  Australia | HBPR: 80  OPR: 86 | NR | **At 12 months**  HBPR: 17 (21)  OPR: 29 (34)  p = 0.07 | **At 12 months**  HBPR: 28 (35)  OPR: 37 (43)  p = 0.29 | **At 12 months**  *Overall:*  HBPR: 0.0 (0.0- 3.7)  OPR: 0.0 (0-6.2)  p = 0.28  *For COPD admissions:*  HBPR: 0 (0-0)  OPR: 0 (0-5)  p = 0.15 | NR |
| Mendes de Oliveira 2010  Brazil | HBPR: 42  OPR: 46 | NR | NR | NR | NR | NR |
| Nolan 2019  UK | HBPR: 154  OPR: 154 | NR | NR | NR | NR | NR |
| Chaplin 2017  UK | HBPR: 51  OPR: 52 | NR | NR | NR | NR | NR |
| Notes: Usual care: patients were managed by their GP, specialist or both according to local practices  COPD: chronic obstructive pulmonary disease; ER: emergency room; HBPR: home-based pulmonary rehabilitationIQR: interquartile range; NR: not reported; OPR: outpatient pulmonary rehabilitation; PR: pulmonary rehabilitation | | | | | | |

| Supplementary Appendix 14. Mental health | | | | | | | | | | | | |  |
| --- | --- | --- | --- | --- | --- | --- | --- | --- | --- | --- | --- | --- | --- |
| **Study** | **Sample size** | **PR duration** | **Time** | **HADS anxiety** | | | **HADS depression** | | | **Others** | | | |
|  |  |  |  | **HBPR**  **Mean±SD** | **Comparison group**  **Mean±SD** | **Diff. between groups**  **MD(95%CI)** | **HBPR**  **Mean±SD** | **Comparison group**  **Mean±SD** | **Diff. between groups**  **MD(95%CI)** | **HBPR**  **Mean±SD** | **Comparison group**  **Mean±SD** | **Diff. between groups**  **MD(95%CI)** | |
| ***HBPR vs usual care*** | | | | | | | | | | | | | |
| Lahham 2019  Australia | HBPR: 29  Usual care: 29 | Active PR phase: 2 months  Maintenance: monthly calls for 6 months | NR | NR | NR | NR | NR | NR | NR | NR | NR | NR | |
| Coultas 2018  USA | HBPR: 149  Usual care: 156 | Active PR phase: 6 months  Maintenance: 10 months | NR | NR | NR | NR | NR | NR | NR | NR | NR | NR | |
| Li 2018  China | HBPR: 82  Usual care: 69 | Active PR phase: 6 months  Maintenance: weekly calls for 6 months | Baseline | NR | NR | NR | NR | NR | NR | **BDI**  HBPR(n=82): 8.4±5.3 | **BDI**  OPR(n=69): 8.4±4.4 | NR | |
|  |  |  | 12 months  *Diff. within groups* | NR | NR | NR | NR | NR | NR | HBPR: 8.0±5.4  p <0.001 | OPR: 7.8±4.4  p <0.001 | NR | |
| Khoshkesht 2015  Iran | HBPR: 35  Usual care: 35 | Active PR phase: 2 months | NR | NR | NR | NR | NR | NR | NR | NR | NR | NR | |
| Pradella 2015  Brazil | HBPR: 32  Usual care: 18 | Active PR phase: 2 months | NR | NR | NR | NR | NR | NR | NR | NR | NR | NR | |
| De Sousa Pinto 2014  Spain | HBPR: 29  Usual care: 21 | Active PR phase: 3 months | NR | NR | NR | NR | NR | NR | NR | NR | NR | NR | |
| Liu 2013  China | HBPR: 30  Usual care: 30 | Active PR phase: 4 months | NR | NR | NR | NR | NR | NR | NR | NR | NR | NR | |
| Mendes de Oliveira 2010  Brazil | HBPR: 42  Usual care: 29 | Active PR phase: 3 months | NR | NR | NR | NR | NR | NR | NR | NR | NR | NR | |
| Moore 2009  UK | HBPR: 14  Usual care: 13 | Active PR phase: 1.5 months | NR | NR | NR | NR | NR | NR | NR | NR | NR | NR | |
| Lalmolda 2017  Spain | HBPR: 21  Usual care: 29 | Active PR phase: 2 months  Maintenance: monthly calls for 12 months | NR | NR | NR | NR | NR | NR | NR | NR | NR | NR | |
| ***HBPR vs OPR*** | | | | | | | | | | | | | |
| Horton 2017  UK | HBPR: 145  OPR: 142 | Active PR phase: 2 months | Baseline | HBPR(n=137): 7.4±4.0 | OPR(n=129): 7.9±4.0 | NR | HBPR(n=137): 6.1±3.6 | OPR(n=129): 5.5±3.1 | NR | NR | NR | NR | |
|  |  |  | 2 months | HBPR(n=81): 7.1±4.0 | OPR(n=86): 6.7±3.8 | 0.7 (0.2, 1.6)  p =0.11 | HBPR(n=82): 6.1±3.7 | OPR(n=86): 5.5±3.2 | 0.8 (0.0, 1.6)  p = 0.04 | NR | NR | NR | |
|  |  |  | 6 months | HBPR(n=68): 7.3±4.3 | OPR(n=73): 8.0±4.2 | 0.1(−0.9, 1.2)  p =0.81 | HBPR(n=68): 6.3±3.6 | OPR(n=73): 6.5±3.6 | 0.1 (−0.9, 1.1)  p = 0.88 | NR | NR | NR | |
| Holland 2017  Australia | HBPR: 80  OPR: 86 | Active PR phase: 2 months | NR | NR | NR | NR | NR | NR | NR | NR | NR | NR | |
| Mendes de Oliveira 2010  Brazil | HBPR: 42  OPR: 46 | Active PR phase: 3 months | NR | NR | NR | NR | NR | NR | NR | NR | NR | NR | |
| Nolan 2019  UK | HBPR: 154  OPR: 154 | Active PR phase: 2 months | NR | NR | NR | NR | NR | NR | NR | NR | NR | NR | |
| Chaplin 2017  UK | HBPR: 51  OPR: 52 | Active PR phase: around 3 months | Baseline | HBPR: 7.9±4.8 | OPR: 7.1±5.0 | NR | HBPR: 6.4±3.8 | OPR: 5.8±3.6 | NR | NR | NR | NR | |
|  |  |  | At PR completion | HBPR(n=22): NR | OPR(n=40): NR | NR  p >0.05 | HBPR(n=22): NR | OPR(n=40): NR | NR  p >0.05 | NR | NR | NR | |
| Notes: Usual care: patients were managed by their GP, specialist or both according to local practices. BDI: Beck depression inventory; CI: Confidence interval; COPD: chronic obstructive pulmonary disease HADS: hospital anxiety and depression scale; HBPR: home-based pulmonary rehabilitationMD: Mean difference; NA: not applicable; NR: not reported; OPR: outpatient pulmonary rehabilitation; PR: pulmonary rehabilitation  SD: standard deviation | | | | | | | | | | | | | |

| Supplementary Appendix 15. Self-efficacy | | | | | | | | | |
| --- | --- | --- | --- | --- | --- | --- | --- | --- | --- |
| **Study** | **Sample size** | **PR duration** | **Time** | **PRAISE** | | | **CSES** | | |
|  |  |  |  | **HBPR**  **Mean±SD** | **Comparison group**  **Mean±SD** | **Diff. between groups**  **MD(95%CI)** | **HBPR**  **Mean±SD** | **Comparison group**  **Mean±SD** | **Diff. between groups**  **MD(95%CI)** |
| ***HBPR vs usual care*** | | | | | | | | | |
| Lahham 2019  Australia | HBPR: 29  Usual care: 29 | Active PR phase: 2 months  Maintenance: monthly calls for 6 months | NR | NR | NR | NR | NR | NR | NR |
| Coultas 2018  USA | HBPR: 149  Usual care: 156 | Active PR phase: 6 months  Maintenance: 10 months | NR | NR | NR | NR | NR | NR | NR |
| Li 2018  China | HBPR: 82  Usual care: 69 | Active PR phase: 6 months  Maintenance: weekly calls for 6 months | NR | NR | NR | NR | NR | NR | NR |
| Khoshkesht 2015  Iran | HBPR: 35  Usual care: 35 | Active PR phase: 2 months | Baseline | NR | NR | NR | **Total score:**  HBPR(n=34): 97.4±20.5  **Negative affect:**  HBPR(n=34): 38.0±6.5  **Intense emotional arousal:**  HBPR(n=34): 25.6± 5.4  **Physical exertion:**  HBPR (n=34): 10.9±3.7  **Weather environmental:**  HBPR(n=34): 16.6±3.3  **Behavioral risk factor**  HBPR(n=34): 6.2±2.7 | **Total score:**  Usual care(n=32): 99.5±17.0  **Negative affect:**  Usual care(n=32): 49.3±3.5  **Intense emotional arousal:**  Usual care(n=32): 24.6±4.4  **Physical exertion:**  Usual care(n=32): 18.0±2.7  **Weather environmental:**  Usual care(n=32): 23.6±2.7  **Behavioral risk factor**  Usual care(n=32): 11.9±1.7 | NR |
|  |  |  | 2 months | NR | NR | NR | **Total score:**  HBPR(n=34): 137.8±10.7  **Negative affect:**  HBPR(n=34): 38.7±5.8  **Intense emotional arousal:**  HBPR(n=34): 27.3±7.0  **Physical exertion:**  HBPR(n=34): 11.5±3.2  **Weather environmental:**  HBPR(n=34): 17.2±2.9  **Behavioral risk factor**  HBPR(n=34): 6.7±3.1 | **Total score:**  Usual care(n=32): 92.5±16.8  **Negative affect:**  Usual care(n=32): 36.4±5.5  **Intense emotional arousal:**  Usual care(n=32): 18.0±2.7  **Physical exertion:**  Usual care(n=32): 10.2±3.0  **Weather environmental:**  Usual care(n=32): 16.22±3.11  **Behavioral risk factor**  Usual care(n=32): 5.6±2.4 | **Total score:** NR  p <0.001  **Negative affect:** NR  p <0.001  **Intense emotional arousal:** NR  p <0.001  **Physical exertion:** NR  p <0.001  **Weather environmental:** NR  p <0.001  **Behavioral risk factor:** NR  p <0.001 |
| Pradella 2015  Brazil | HBPR: 32  Usual care: 18 | Active PR phase: 2 months | NR | NR | NR | NR | NR | NR | NR |
| De Sousa Pinto 2014  Spain | HBPR: 29  Usual care: 21 | Active PR phase: 3 months | NR | NR | NR | NR | NR | NR | NR |
| Liu 2013  China | HBPR: 30  Usual care: 30 | Active PR phase: 4 months | NR | NR | NR | NR | NR | NR | NR |
|  |  |  |  |  |  |  |  |  |  |
|  |  |  |  |  |  |  |  |  |  |
| Mendes de Oliveira 2010  Brazil | HBPR: 42  Usual care: 29 | Active PR phase: 3 months | NR | NR | NR | NR | NR | NR | NR |
| Moore 2009  UK | HBPR: 14  Usual care: 13 | Active PR phase: 1.5 months | NR | NR | NR | NR | NR | NR | NR |
| Lalmolda 2017  Spain | HBPR: 21  Usual care: 29 | Active PR phase: 2 months  Maintenance: monthly calls for 12 months | NR | NR | NR | NR | NR | NR | NR |
| ***HBPR vs OPR*** | | | | | | | | | |
| Horton 2017  UK | HBPR: 145  OPR: 142 | Active PR phase: 2 months | Baseline | HBPR (n=130): 47.2±8.1 | OPR (n=122): 44.8±7.0 | NR | NR | NR | NR |
|  |  |  | 2 months | HBPR(n=81): 44.7±8.5 | OPR(n=84): 47.1±7.7 | −2.6 (−4.9, −0.3)  p = 0.03 | NR | NR | NR |
|  |  |  | 6 months | HBPR(n=68): 43.2±8.9 | OPR(n=72): 44.1±8.8 | −1.1 (−4.0, 1.7)  p = 0.42 | NR | NR | NR |
| Holland 2017  Australia | HBPR: 80  OPR: 86 | Active PR phase: 2 months | Baseline | HBPR: 48±7 | OPR: 46±9 | NR | NR | NR | NR |
|  |  |  | 2 months  *Diff. within groups from baseline*  *MD(95%CI)* | HBPR(n=72): NR  *1.1(-0.7, 2.9)*  *p=NR* | OPR(n=76): NR  *-0.2(-1.9, 1.6)*  *p=NR* | 1.2 (-1.2, 3.7)  p = NR | NR | NR | NR |
|  |  |  | 12 months  *Diff. within groups from baseline*  *MD(95%CI)* | HBPR(n=72): NR  *1.7(-0.2, 3.7)*  *p = NR* | OPR(n=76): NR  *2.4(0.4, 4.3)*  *p = NR* | -0.6(-3.4, 2.1)  p = NR | NR | NR | NR |
| Mendes de Oliveira 2010  Brazil | HBPR: 42  OPR: 46 | Active PR phase: 3 months | NR | NR | NR | NR | NR | NR | NR |
| Nolan 2019  UK | HBPR: 154  OPR: 154 | Active PR phase: 2 months | NR | NR | NR | NR | NR | NR | NR |
| Chaplin 2017  UK | HBPR: 51  OPR: 52 | Active PR phase: around 3 months | Baseline | HBPR: 45.6±7.7 | OPR: 45.7±7.7 | NR | NR | NR | NR |
|  |  |  | At program completion | HBPR: NR | OPR: NR | NR  p > 0.05 | NR | NR | NR |
| Notes: Usual care: patients were managed by their GP, specialist or both according to local practices.  CI: Confidence interval; COPD: chronic obstructive pulmonary disease; CSES: COPD self-efficacy scale; HBPR: home-based pulmonary rehabilitationMD: Mean difference; NR: not reported; OPR: outpatient pulmonary rehabilitation; PR: pulmonary rehabilitation; PRAISE: Pulmonary Rehabilitation Adapted Index of Self-Efficacy; SD: standard deviation | | | | | | | | | |
